# Examining the Impact of Acute Exercise and Arousal Reappraisal on Stressor‐Evoked Psychological and Cardiovascular Responses

**DOI:** 10.1111/psyp.70353

**Published:** 2026-06-28

**Authors:** Taryn E. Cook, Sarah E. Williams, Thomas A. Fergus, Annie T. Ginty

**Affiliations:** ^1^ Department of Psychology and Neuroscience Baylor University Waco Texas USA; ^2^ School of Sport, Exercise, and Rehabilitation Sciences University of Birmingham Birmingham UK

## Abstract

Extreme stressor‐evoked psychophysiological responses are associated with adverse health outcomes. The present study examined the individual and combined influence of exercise and arousal reappraisal on stressor‐evoked psychophysiological responses. Participants (*N* = 238) were randomly assigned to one of four groups: control (CTRL), arousal reappraisal only (AR), exercise only (EX), or combined arousal reappraisal and exercise (AR + EX). After completing baseline 1, the assigned experimental condition, recovery, and baseline 2, participants underwent a speech task. Cardiovascular measures were obtained during baselines and the stress task, and state psychological measures were obtained after informed consent and after the stress task. Trait reappraisal was also measured. There were no group differences in stressor‐evoked cardiovascular responses. However, despite similar stress intensity ratings across all groups, participants in the AR group interpreted their stress more positively. Additionally, moderation analyses demonstrated that individuals with higher trait reappraisal in the EX and AR + EX groups viewed their physiological arousal as more helpful than individuals in the CTRL group. Arousal reappraisal may improve interpretations of acute stress. Additionally, acute exercise may be most beneficial for reducing negative interpretations of perceived physiological arousal when trait reappraisal levels are high. Future research should explore whether repeated arousal reappraisal and exercise training may promote more adaptive stressor‐evoked responses.

## Impact Statement

Our findings add a new perspective on previous work by examining the individual and combined effects of arousal reappraisal and exercise on both physiological and psychological responses to acute stress. While there were no group differences in stressor‐evoked cardiovascular responses, we provide strong evidence that both arousal reappraisal and exercise may improve interpretations of stress.

## 1 Introduction

Psychological stress is widespread (American Psychological Association 2023; Dave et al. 2024; Piao et al. 2024) and has been associated with a range of adverse physical and mental health outcomes (e.g., Agorastos and Chrousos 2022; Cohen et al. 2016, 2007; Epel et al. 2018; Kivimäki et al. 2023; McFarlane 2010; Steptoe and Kivimäki 2012). Psychological stress activates physiological responses that prepare the individual to confront or escape perceived threats. In the short‐term, these acute responses to stress are adaptive as they facilitate the mobilization of energy and resources necessary for active coping strategies (e.g., fight‐or‐flight behaviors; for reviews see: Gianaros and Jennings 2018; Ginty et al. 2017; Obrist 1981; Schneiderman et al. 2005; Whittaker et al. 2021). Yet over time, chronic activation may contribute to a range of negative health outcomes (for reviews see: Gianaros and Jennings 2018; Kivimäki et al. 2023; Schneiderman et al. 2005; Turner et al. 2020; Vaccarino and Bremner 2024; Whittaker et al. 2021). However, as stress cannot be entirely avoided (Crum et al. 2013; Rudland et al. 2020; Souza‐Talarico et al. 2016), examining methods for improving interpretations of stress may be beneficial in disrupting the relationship between psychological stress and poor health outcomes (Kivimäki et al. 2023; Vaccarino and Bremner 2024).

One potential strategy for improving interpretations of stress is arousal reappraisal, which involves reframing the physiological sensations of a stress response (e.g., heart racing) as helpful or facilitative for performance rather than harmful (Jamieson, Hangen, et al. 2018; Jamieson et al. 2013; Seery 2013). The effects of arousal reappraisal training on physiological responses to acute stress are mixed, with a recent meta‐analysis finding no overall differences in physiological responses between arousal reappraisal and control participants (Liu et al. 2019). However, given that arousal reappraisal is focused on reframing physiological sensations and not eliminating them, the lack of relationship is not surprising. Indeed, previous work has found that arousal reappraisal improves psychological responses to acute stress in both the laboratory and real‐world contexts, such as a classroom (Beltzer et al. 2014; Gurera and Isaacowitz 2022; Huang et al. 2023; Jacquart et al. 2020; Jamieson et al. 2022, 2016; Liu et al. 2019; Sammy et al. 2017; Sharpe et al. 2024). Consequently, arousal reappraisal may be an effective way to elicit more positive perceptions of stress.

Although the extant literature indicates that arousal reappraisal improves psychological responses to stress, a limited range of psychological outcomes have been explored, with the most common being stressor‐evoked anxiety (for review see: Liu et al. 2019). As arousal reappraisal consists of reinterpreting, rather than suppressing or ignoring, the physiological sensations of stress (Jamieson et al. 2013), measuring both the intensity and interpretation of perceived physiological arousal, rather than anxiety, may illuminate how arousal reappraisal promotes more adaptive stress responses (Ginty et al. 2022).

Another possible strategy for altering individual physiological and psychological stress responses is through engaging in exercise (Gerber et al. 2014; Hamer et al. 2012; Nguyen‐Michel et al. 2006). Laboratory studies have repeatedly demonstrated that acute exercise can reduce blood pressure (BP) responses to a subsequent acute psychological stress task (for reviews see: Chen et al. 2022; Hamer et al. 2006; Mariano et al. 2022; Morava et al. 2024). Previous work has suggested that the reduction in BP responses to stress may be due to decreases in sympathetic nervous system activity and increases in vasodilation that occur in response to acute exercise (Hamer et al. 2006; Morava et al. 2024). However, no consistent relationship between acute exercise and stressor‐evoked heart rate (HR) reactivity has emerged, likely due to differences in methodology across studies (Ginty et al. 2026). Similarly, results are also mixed for studies examining the effects of acute exercise on psychological responses to acute stress, with much of the existing work reporting no impact of exercise on stressor‐evoked anxiety or perceived stress (for review see: Morava et al. 2024). However, most studies measuring psychological responses to stress only examined the intensity of these psychological responses, not their interpretation (e.g., LaManca et al. 2001; Leow et al. 2021; Szabo et al. 1993; Wheeler et al. 2019). One study that assessed both intensity and interpretation of self‐confidence during an acute psychological stress task found that participants who completed the yoga session, but not those in the control group, interpreted their self‐confidence as helpful for task performance, indicating exercise may alter interpretation of psychological responses to stress (Benvenutti et al. 2017). To our knowledge, there has been limited work examining the effects of cardiorespiratory exercise on interpretations of stressor‐evoked psychological responses.

As lack of time and accessibility to equipment are often reported as an obstacle to physical activity (Ashton et al. 2017; Ebben and Brudzynski 2008; Gibala 2007), high intensity interval training (HIIT), which provides health benefits at shorter durations than steady‐state exercise and requires no equipment, may be a useful exercise modality for improving perceptions of stress. Limited research has examined how HIIT may improve psychophysiological responses to acute stress. One study in a sample of males found both HIIT and moderate‐intensity exercise attenuated BP reactivity, but not HR reactivity, to stress compared to a control group (Farah et al. 2021). Contrastingly, a separate study found only moderate‐intensity exercise, not HIIT, attenuated HR reactivity, but found that neither type of exercise impacted BP reactivity to a passive stressor (Meireles et al. 2020). Neither study examined psychological responses to acute stress.

It is possible that combining exercise and arousal reappraisal may promote more adaptive stressor‐evoked responses than either technique alone. Building on prior work that indicates repeated exposure to vigorous exercise promotes physiological adaptations to psychological stress and reduces anxiety sensitivity, combining exercise with arousal reappraisal may offer individuals exposure to stress‐related sensations and then explicit guidance on reinterpreting those sensations which could then be applied to other stressors (LeBouthillier and Asmundson 2015; Smits et al. 2008; Sothmann 2006; Sothmann et al. 1996; Stubbs et al. 2017). To our knowledge, only one study has examined how combining exercise and arousal reappraisal impacts psychological responses to acute stress. Jacquart et al. (2020) randomized 167 participants with depressive symptoms to one of four groups: control, exercise, arousal reappraisal, or combined exercise and arousal reappraisal. Using paired contrasts, they found that the combined group had more adaptive psychological responses than the exercise and control groups, and the reappraisal group reported more adaptive psychological responses than the control group. There were no differences between the combined group versus the reappraisal only group. Notably, physiological responses to stress were not reported in the manuscript, so the effect of combining reappraisal and exercise on stressor‐evoked physiological reactivity is unknown.

The current study sought to extend the work of Jacquart et al. (2020) by (1) utilizing a generally healthy sample rather than recruiting those with high depressive symptoms, (2) assessing both physiological and psychological responses, and (3) directly comparing all groups within a statistical model, rather than relying only on paired contrasts. The aim of the current study was to examine the effects of arousal reappraisal alone, acute exercise alone, and arousal reappraisal combined with acute exercise on psychophysiological responses to acute stress. Participants were randomly assigned to one of four groups: control (CTRL), arousal reappraisal only (AR), acute exercise only (EX), and combined arousal reappraisal and acute exercise (AR + EX). It was hypothesized that individuals in the AR, EX, and AR + EX groups would demonstrate more adaptive psychophysiological responses to the acute stressor than the CTRL group, reflected in lower stressor‐evoked blood pressure responses, along with reduced perceived stress and physiological arousal intensity, and more positive interpretations of perceived stress and physiological arousal. We further hypothesized that the AR + EX group would exhibit the most favorable responses with lower blood pressure responses and improved psychological responses to acute stress than either the AR group or EX group alone. Based on prior work, no significant differences in stressor‐evoked heart rate responses were anticipated (Griffin and Howard 2021; Hamer et al. 2006; Liu et al. 2019; Morava et al. 2024). On an exploratory basis, we examined whether the relationships between stressor‐evoked psychological responses and group assignment were moderated by trait reappraisal, as prior work has shown that the effectiveness of interventions may depend on individuals' innate reappraisal abilities (Mauersberger et al. 2018).

## 2 Methods

### 2.1 Transparency and Openness

This study's design and analyses were pre‐registered on the Open Science Framework during data collection, prior to data entry and analysis; see: https://doi.org/10.17605/OSF.IO/GCT2Q. A priori power analyses (*α* = 0.05, power = 0.80, small to medium effect size) using G*Power suggested a minimum of 192 participants was needed to detect small to medium effect sizes for the Group × Time interactions. A small to medium effect size was selected based on prior meta‐analytic work examining psychological and physiological responses to either acute exercise or cognitive reappraisal (Chen et al. 2022; Hamer et al. 2006; Liu et al. 2019; Mariano et al. 2022). The sample size aligns with previous experimental studies (Jacquart et al. 2020; Thomas and Kamarck 2023). Our pre‐registration indicated we would test a minimum of 192 participants and that we would recruit up to 300 participants or until the end of May 2025 (whichever came first).

### 2.2 Participants

Participants were 250 healthy young adults recruited using the university's online psychology subject pool (SONA systems) and from the community via flyers and online advertisements. Inclusion criteria included: being an adult under the age of 40, not currently pregnant, and not prohibited from engaging in physical exercise. Data were missing from 12 participants^1^ due to the following reasons: withdrew during exercise due to feeling unwell or being unable to maintain prescribed speed (*n* = 5); withdrew during the acute psychological stress task due to self‐reported extreme distress (*n* = 4); withdrew due to lightheadedness during blood pressure measurements during baseline 1 (*n* = 1); after providing informed consent and beginning the study, the participant disclosed to research staff they had completed exercise within the past 12 h, thus making them ineligible (*n* = 1); and data were unusable due to a research assistant error while implementing exercise intensity during sprints (*n* = 1). Independent sample *t‐*tests and chi‐square analyses demonstrated no statistically significant differences between those with unusable data and the rest of the sample on age (*p* = 0.586), biological sex (*p* = 0.904), and race (*p* = 0.640). The final usable sample size was 238 participants (mean age = 18.93, SD = 1.10 years; 60.1% female; 42.0% non‐Hispanic White; 20.6% Hispanic or Latino). Participants were asked to refrain from: drinking alcohol and engaging in vigorous exercise for 12 h, drinking caffeine for 2 h, and consuming food or any other drink (except water) for 2 h prior to their appointment time. Participants chose to receive either 3 h of SONA research credits applied to their psychology and neuroscience courses or a $25 Amazon gift card. All participants provided written informed consent prior to data collection. The study was conducted in accordance with the Declaration of Helsinki and was approved by the university's Institutional Review Board. Data collection occurred between September 2024 and May 2025.

### 2.3 Procedure Overview

**FIGURE 1:**
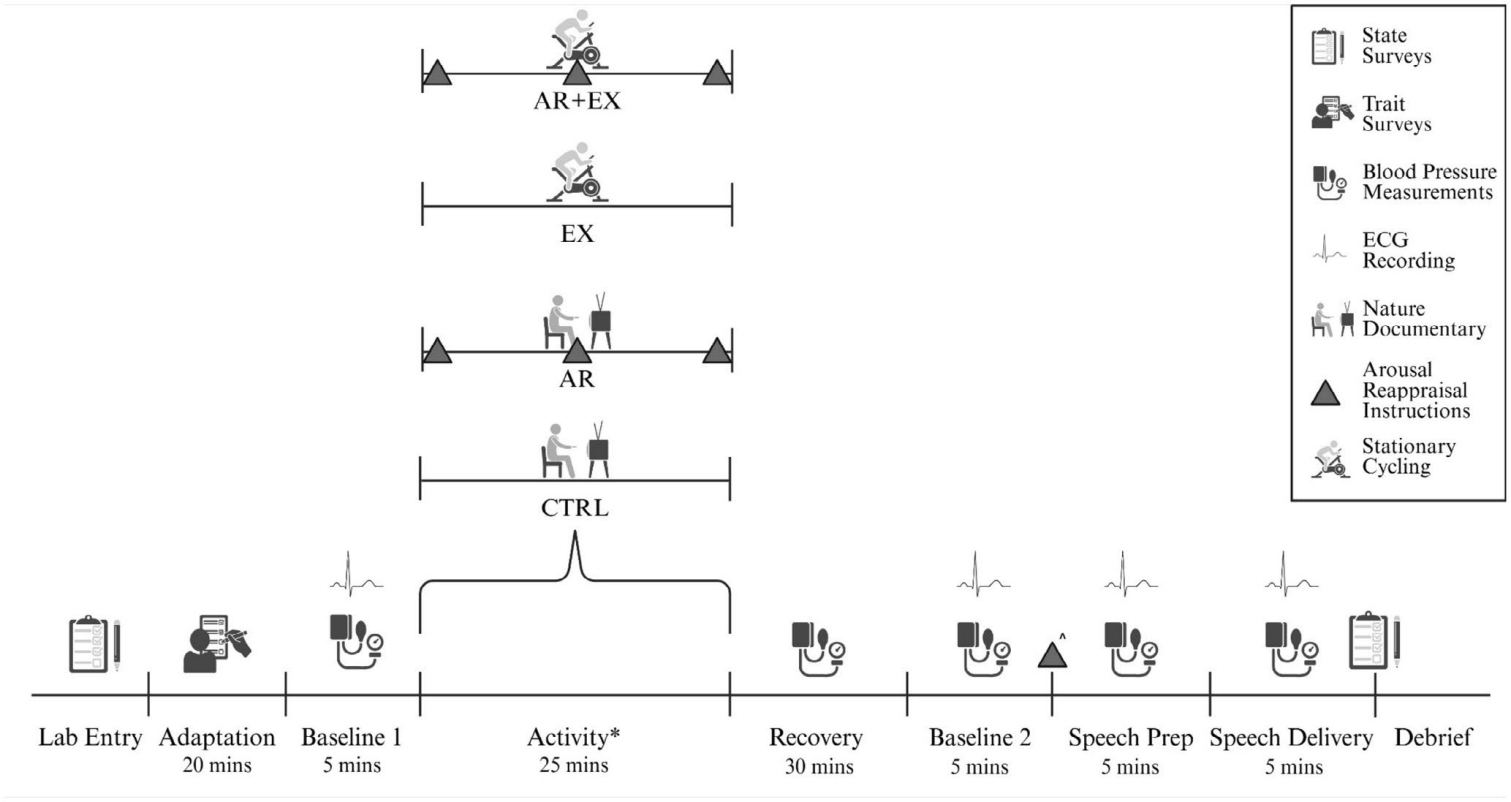
Visual depiction of study procedure. AR, arousal reappraisal only group; AR + EX, combined arousal reappraisal and exercise group; CTRL, control group; EX, exercise only group. *Participants were randomly assigned to one of the four activity groups. ^^^Only participants in the AR + EX and AR groups received reappraisal instructions before the speech preparation period.

See Figure 1 for the full protocol. All participants completed a single laboratory visit lasting approximately 2.5 h. Upon arrival to the laboratory, participants provided informed consent and were then randomly assigned to one of four groups (CTRL, AR, EX, AR + EX). Randomization was completed using a block design (blocks of 4) and stratified by gender. Participants then completed the state questionnaires^2^ and had anthropometric measurements taken. Next, participants were fitted with physiological equipment (i.e., BP cuff, electrocardiogram electrodes, and heart rate chest strap). Following a 20‐min adaptation period where participants completed demographic and trait questionnaires, participants completed a formal 5‐min baseline during which cardiovascular measurements were obtained. After the BP cuff was removed, participants completed one of four conditions for the 25‐min activity phase (see Activity Phase description below for details). Immediately following the activity, the BP cuff was placed back on participants. Participants engaged in a 30‐min recovery phase, where all participants watched a neutral documentary (different from the documentary used for CTRL and AR groups during the activity phase). A neutral documentary was selected for the 30‐min recovery period based on previous research demonstrating idleness may be aversive and may increase boredom and anxiety (Yang and Hsee 2019). Next, participants completed another 5‐min formal baseline. Following the second baseline, participants listened to instructions for the speech task, completed a 5‐min speech preparation period, and then completed a 5‐min speech delivery period. Immediately following the speech delivery, participants completed the state questionnaires and had physiological equipment removed. Participants were then debriefed.

### 2.4 Activity Phases for Current Study

#### 2.4.1 Control

Participants assigned to a non‐exercise group (CTRL, AR) watched 25 min of a neutral nature documentary.

#### 2.4.2 Exercise

**FIGURE 2:**
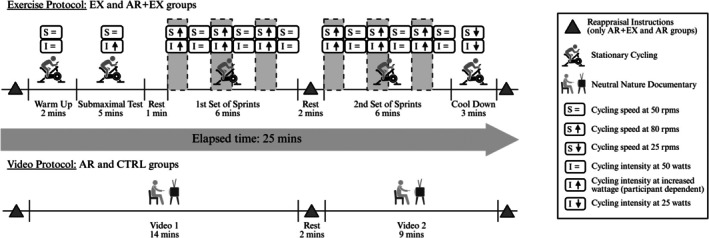
Visual depiction of activity phase. AR, arousal reappraisal only group; AR + EX, combined arousal reappraisal and exercise group; CTRL, control group; EX, exercise only group.

Participants assigned to the exercise groups (EX, AR + EX) completed a 25‐min exercise paradigm (see Figure 2) on a standard upright bicycle ergometer (Corival CPET or Monark 928E).^3^ The exercise paradigm was selected based off previous work examining the impact of acute exercise on cardiovascular stress reactivity (Aladro‐Gonzalvo et al. 2019). During exercise, HR was recorded discontinuously and closely monitored in real‐time via a Polar H10 HR monitor (Polar Electro, Finland) to ensure adequate exertion during exercise. After the 2‐min warmup, participants completed a 5‐min submaximal exercise test, where the resistance was increased every minute until the participant reached at least 60% of their heart rate reserve (HRR). 60% HRR was calculated as ([220 − age − resting HR] × 0.60) + resting HR, where resting HR was their lowest HR during baseline 1 (American College of Sports Medicine 2013). The resistance at which participants reached 60% HRR was used as the sprinting resistance for the interval portions moving forward. Following the 5‐min submaximal exercise test, participants received a 1‐min break. Participants then completed 6‐min of cycling, alternating between 30 s of sprinting and 30 s of recovery cycling. During the sprint portions, participants cycled at the resistance determined during the submaximal test and a speed of 80 rpm (RPM). During the recovery portions, participants cycled at a lower resistance and speed (50 RPM). After a 2‐min break, participants completed another 6‐min bout alternating between sprints and recovery. Participants then completed a 3‐min cooldown. Participants were only allowed to drink water during breaks and were not allowed to listen to music.

#### 2.4.3 Arousal Reappraisal

Participants assigned to the AR group read and listened to arousal reappraisal instructions regarding physiological arousal during stress at three time points during the activity phase (e.g., before the activity began, at minute 14 of the activity period and upon completion of the activity) and then immediately before the stress task (see Supporting Information for full arousal reappraisal instructions). These instructions were adapted from previous reappraisal studies and tested in a pilot study to ensure efficacy (Beltzer et al. 2014; Jamieson et al. 2010, 2016). Each set of instructions presented the information slightly differently but reinforced to participants that while individuals often perceive physiological arousal during stress as negative, increased arousal during stressful situations can improve performance (Jamieson et al. 2012).

#### 2.4.4 AR + EX

Participants in the AR + EX group completed the same exercise paradigm as the EX group and received arousal reappraisal instructions at identical timepoints in the protocol to the AR group. The arousal reappraisal instructions given to the AR + EX group and AR group were identical, except during each instruction, participants in the AR + EX group received additional instructions to think about their HR during exercise.

### 2.5 Acute Psychological Stress Task

A speech task paradigm was used to evoke acute psychological stress. First, all participants were given a prompt to recall and then prepare a speech about a time they either felt most like a failure or when they made a large mistake. Previous research has found this prompt induces feelings of stress and perturbs cardiovascular activity for our sample age range (Tyra, Young, and Ginty 2025). Participants were given 5 min to prepare and 5 min to deliver a speech. To enhance perceptions of stress, two emotionally unresponsive research assistants sat directly across from the participant and the participant was told that their speech was being videotaped for performance evaluation at a later timepoint (participants were not actually video recorded; Dickerson and Kemeny 2004; Kirschbaum et al. 1993; Kirschbaum et al. 1992).

### 2.6 Measures

#### 2.6.1 Cardiovascular Measurements

Cardiovascular measurements were obtained throughout the laboratory visit. Systolic and diastolic blood pressure (SBP, DBP) were measured discontinuously every minute during baseline 1, baseline 2, speech preparation, and speech delivery utilizing a standard, automatic sphygmomanometer (Carescape, V100, General Electric, El Paso, TX, USA). HR was measured continuously during baseline 1, baseline 2, speech preparation, and speech delivery using a mobile electrocardiography (ECG) device (Bio Protech Inc., Chino, CA). Data were collected at 500 Hz and then automatic R‐peak detection and manual removal of artifacts were completed with MindWare's HR/HRV analysis software. ECG data were then uploaded to Kubios HRV. HR was also continuously monitored during exercise utilizing the Polar H10 HR monitor (Polar Electro, Finland). Continuously monitored HR was used to adjust the cycling wattage in real time to ensure participants were exercising at the appropriate rate. Participants in the non‐exercise groups (CTRL, AR) also wore the Polar H10 HR monitor for consistency. Speech preparation and speech delivery were treated as one continuous stress task for data analysis (e.g., Brindle et al. 2016; Tyra et al. 2020; Tyra, Garner, and Ginty 2025). SBP, DBP, and HR averages were calculated for each participant and resulted in three measures: baseline 1, baseline 2, and stressor.

#### 2.6.2 State Questionnaires

##### 2.6.2.1 Perceived Stress and Perceived Physiological Arousal

Participants rated the intensity and interpretation of their state levels of perceived stress and perceived physiological arousal using four individual items. Separately for perceived stress and perceived arousal, participants first rated the intensity of the feeling (i.e., “How stressed did you feel during the task?” and “How physiologically aroused were during the task?”) on a 7‐point Likert scale (1 = not at all, 7 = extremely). Then participants separately rated their interpretation of these feelings (“Did you regard these feelings of stress as being positive/negative in relation to performance of the task?” and “Did you regard this physiological arousal as being positive or negative in relation to the performance of the task?”) on a 7‐point Likert scale (−3 = very debilitative [negative], 0 = unimportant, 3 = very facilitative [positive]). The perceived stress and physiological arousal items have been frequently used in acute psychological stress research (Trotman et al. 2019; Tyra et al. 2023; Tyra, Young, and Ginty 2025).

##### 2.6.2.2 State Reappraisal

Immediately after the stress task, participants' use of reappraisal during the task was measured with the reappraisal subscale of the state‐modified version of the Emotion Regulation Questionnaire (ERQ: Egloff et al. 2006; Gross and John 2003). The subscale consists of 3 items (e.g., “I tried to see the task as positive as possible”) and participants responded using a six‐point Likert scale (0 = not at all, 5 = extremely), where a higher scale indicates higher levels of reappraisal during the task.

#### 2.6.3 Trait Questionnaires

##### 2.6.3.1 Hospital Anxiety Depression Scale

To examine if there were differences between groups on participant mental health symptoms, participants reported their symptoms of anxiety and depression using the 14‐item Hospital Anxiety and Depression Scale (HADS; Zigmond and Snaith 1983). The anxiety and depression subscales consist of seven items each, with participants responding using a 4‐point Likert scale (0–3) where higher scores indicate greater anxiety or depression. The HADS has demonstrated anxiety and depressive symptom scores to be valid and reliable (Bramley et al. 1988; Herrmann 1997). Internal consistency for the present study was good for the anxiety subscale (Cronbach's *α* = 0.81) and acceptable for the depression subscale (Cronbach's *α* = 0.62).

##### 2.6.3.2 Physical Activity

To examine if there were differences between the groups in physical activity levels, participants answered questions regarding their physical activity (PA) over the past week using the Short‐Form International Physical Activity Questionnaire (SF‐ IPAQ; Craig et al. 2003), which assesses vigorous PA, moderate PA, and walking activity. Based on IPAQ scoring recommendations, individuals were categorized into “high”, “moderate” or “low” levels of PA (see Supporting Information for full scoring details). The SF‐IPAQ is widely used to assess self‐reported PA in young adults (e.g., Abrantes et al. 2022; Alghamdi et al. 2025; Ge et al. 2019) and has demonstrated acceptable validity and reliability (Craig et al. 2003; Murphy et al. 2017).

##### 2.6.3.3 Trait Reappraisal

Trait reappraisal was assessed during the adaptation period. Participants completed the 10‐item Emotion Regulation Questionnaire (ERQ; Gross and John 2003), which consists of two subscales that assess habitual use of suppression (e.g., “I keep my emotions to myself”) and reappraisal (e.g., “When I'm faced with a stressful situation, I make myself think about it in a way that makes me stay calm.”). Participants responded using a seven‐point Likert scale (1 = strongly disagree, 7 = strongly agree), with higher scores for each subscale indicating higher use of suppression or reappraisal. The ERQ has demonstrated reliable and valid scores for both reappraisal and suppression (Gross and John 2003; Sala et al. 2012) In the present study, internal consistency was acceptable for both the reappraisal (Cronbach's *α* = 0.75) and suppression (Cronbach's *α* = 0.70) subscales. In the present study, only the reappraisal subscale was used.

### 2.7 Statistical Analyses

Statistical analysis was conducted in SPSS (IBM Corp, USA, Version 31). As indicated in our pre‐registration, physiological data were analyzed for outliers three standard deviations from the mean (SBP: *n* = 4; DBP: *n* = 3; HR: *n* = 2). Outliers were removed from analyses on a by‐variable basis (e.g., the outliers for SBP were removed only for the SBP analysis). To examine whether groups differed across any continuous variables that may impact variables of interest (i.e., age, BMI, HADS anxiety, HADS depression, and trait reappraisal), a one‐way analysis of variance (ANOVA) was conducted, and means and standard deviations were calculated. For categorical variables that may impact variables of interest (i.e., self‐reported physical activity levels, biological sex and race/ethnicity), chi‐square tests of independence were conducted to examine the relationships between the groups and the variables, and frequencies and percentages were calculated. When significant differences between the groups were present, these variables were then entered as covariates in the subsequent analytic models.

Then, to check the effectiveness of the arousal reappraisal manipulation, a one‐way ANOVA was run to examine whether groups (CTRL, AR, EX, AR + EX) differed in their use of reappraisal during the stress task. To assess if participants, on average, exercised at the correct intensity during the acute exercise, paired samples *t*‐tests (60% HRR predicted for the submaximal test; average % HRR during the sprints of the exercise period) were conducted.

Next, a series of 4 group (CTRL, AR, EX, AR + EX) × 3 time (baseline 1, baseline 2, stress task) ANOVAs were employed to examine group differences in cardiovascular responses to the acute psychological stress task. Baseline 1 occurred before the activity phase and baseline 2 occurred after the activity phase. Similar 4 × 3 ANOVAs were conducted separately for SBP, DBP, and HR. If Mauchley's Test of Sphericity indicated the assumption of sphericity was violated, Greenhouse–Geisser corrected degrees of freedom and *p‐*values were reported. When significant results were observed, post hoc Bonferroni corrected comparisons were conducted.

Then, a series of one‐way ANCOVAs (CTRL, AR, EX, AR + EX) were employed to examine group differences in psychological responses to the acute psychological stress task. Separate ANCOVAs were conducted for perceived stress intensity, perceived stress interpretation, perceived physiological arousal intensity, and perceived physiological arousal interpretation, with baseline levels of each respective variable entered as the covariate. When significant results were observed, post hoc Bonferroni corrected comparisons were conducted.

Lastly, on an exploratory basis, to test if trait reappraisal moderated the relationship between group assignment and psychological responses to acute stress, the SPSS macro PROCESS “Model 1” (version 5.0) was employed (Hayes 2017). The PROCESS macro was run separately for intensity and interpretation of perceived stress and perceived physiological arousal, with baseline levels of each respective variable entered as the covariate.

For all analyses, the alpha level was set at 0.05.

In full transparency, the analyses presented here deviate slightly from those initially proposed. Although the state questionnaires were administered at four points as pre‐registered (after informed consent, after the activity, after recovery, and after the stressor) only two time points were used in the analyses (after informed consent and after the stressor). This decision was made prior to data analyses to allow us to focus on the main aim of the study (i.e., to examine whether acute exercise, arousal reappraisal, or a combination altered responses to acute stress). Additionally, based off the work of Thomas and Kamarck (2023), we decided a priori to examine if groups differed on covariates and only conducted covariate analyses on variables where there were differences across groups. Lastly, given the number of variables included in the pre‐registration, only a subset has been included in the current manuscript to maintain coherence and clarity.

## 3 Results

### 3.1 Participant Characteristics

Participant demographics for the entire sample and by group are reported in Table 1. The groups (CTRL, AR, EX, AR + EX) did not differ significantly on any variables that may impact variables of interest (e.g., age, BMI, biological sex, race/ethnicity, trait reappraisal, HADS anxiety, HADS depression, self‐reported physical activity).

**TABLE 1:** Participant demographics for the total sample and by group.

Participant characteristics	Total	CTRL	AR	EX	AR + EX	*p*
	*N* = 238	*n* = 60 (25.2%)	*n* = 62 (26.1%)	*n* = 60 (25.2%)	*n* = 56 (23.5%)	
Age, years, *M* (SD)	18.93 (1.10)	19.05 (1.37)	18.81 (0.88)	18.88 (1.06)	19.0 (1.04)	0.611
BMI, kg/m^2^, *M* (SD)	24.07 (3.86)	24.06 (4.18)	24.13 (3.94)	24.42 (3.54)	23.64 (3.81)	0.755
Biological sex, *n* (%)						0.992
Female	143 (60.1)	36 (60.0)	37 (60.0)	37 (61.7)	33 (58.9)	
Male	95 (39.9)	24 (40.0)	25 (40.0)	23 (38.3)	23 (41.1)	
Race/Ethnicity, *n* (%)						0.664
Hispanic/Latino	49 (20.6)	11 (18.3)	10 (16.1)	13 (21.7)	15 (26.8)	
Asian	34 (14.3)	8 (13.3)	11 (17.7)	8 (13.3)	7 (12.5)	
Black or African American	30 (12.6)	8 (13.3)	6 (9.7)	11 (18.3)	5 (8.9)	
Non‐Hispanic White	100 (42.0)	25 (41.7)	28 (45.2)	26 (43.4)	21 (37.5)	
Other	6 (2.5)	1 (1.7)	3 (4.8)	0 (0.0)	2 (3.6)	
More than one	19 (8.0)	7 (11.7)	4 (6.5)	2 (3.3)	6 (10.7)	
Trait reappraisal, *M* (SD)	5.02 (0.84)	4.89 (0.78)	5.08 (0.85)	5.07 (0.75)	5.04 (0.97)	0.576
HADS anxiety, *M* (SD)	7.55 (3.91)	7.57 (4.28)	7.50 (4.05)	7.37 (3.25)	7.80 (4.08)	0.946
HADS depression, *M* (SD)	3.28 (2.39)	3.32 (2.84)	3.34 (2.14)	3.17 (2.23)	3.31 (2.35)	0.979
Physical activity, *n* (%)						0.922
High	157 (70.0)	36 (60.0)	43 (74.2)	40 (66.7)	38 (67.9)	
Moderate	49 (20.6)	13 (21.7)	13 (21.0)	13 (21.67)	10 (17.9)	
Low	7 (2.9)	1 (1.7)	1 (1.6)	3 (5.0)	2 (3.6)	
Missing	25 (10.5)	10 (16.7)	5 (8.1)	4 (6.7)	6 (10.7)	

*Note:* *p*‐Values indicated no significant differences between groups as determined by one‐way ANOVAs or chi‐square tests. Missing data for physical activity are due to participants stating they did not know the time spent on different types of physical activity.

Abbreviations: AR, arousal reappraisal only group; AR + EX, combined arousal reappraisal and exercise group; BMI, body mass index; CTRL, control group; EX, exercise only group; HADS, Hospital Anxiety and Depression Scale.

### 3.2 State Reappraisal Manipulation Check

**FIGURE 3:**
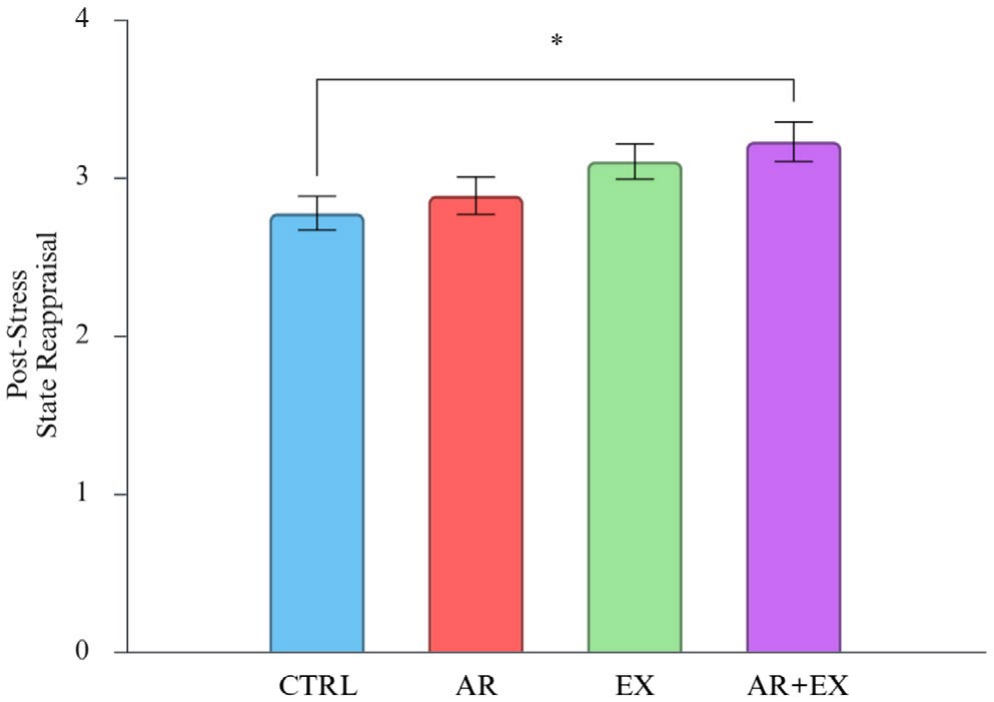
Mean post‐stress state reappraisal values by group. Error bars represent standard error of means. * indicates a statistically significant difference between the CTRL and AR + EX groups. AR, arousal reappraisal only group; AR + EX, combined arousal reappraisal and exercise group; CTRL, control group; EX, exercise only group.

There were statistically significant differences between groups on average state reappraisal score during the acute psychological stress task, *F*(3, 232) = 3.14, *p* = 0.026, *η*_p_^2^ = 0.04. Post hoc comparisons indicated that the AR + EX group (*M* = 3.23, SD = 0.93) reported significantly higher state reappraisal use during the stress task than the CTRL group (*M* = 2.77, SD = 0.82, *p* = 0.031; see Figure 3).

### 3.3 Exercise Intensity Manipulation Check

There were no statistically significant differences between estimated 60% HRR (EX group average estimated 60% HRR HR, *M* = 148.05, SD = 4.83 bpm; EX + AR group averaged estimated 60% HRR HR, *M* = 148.00, SD = 4.69 bpm) and average HR during the sprints (EX group average HR, M = 148.30, SD = 13.67 bpm; EX + AR group average HR, *M* = 150.73, SD = 15.13 bpm) for either the EX group or AR + EX group (*p*s ≥ 0.136), indicating participants, on average, exercised at the correct intensity during the acute exercise. During the sprints, the average %HRR was *M* = 60.38, SD = 8.27 for the EX group and *M* = 62.42, SD = 10.03 for the EX + AR group.

### 3.4 Cardiovascular Analyses

Mean cardiovascular values for SBP, DBP, and HR for each group and phase are displayed in Table S1.

#### 3.4.1 Systolic Blood Pressure

**FIGURE 4:**
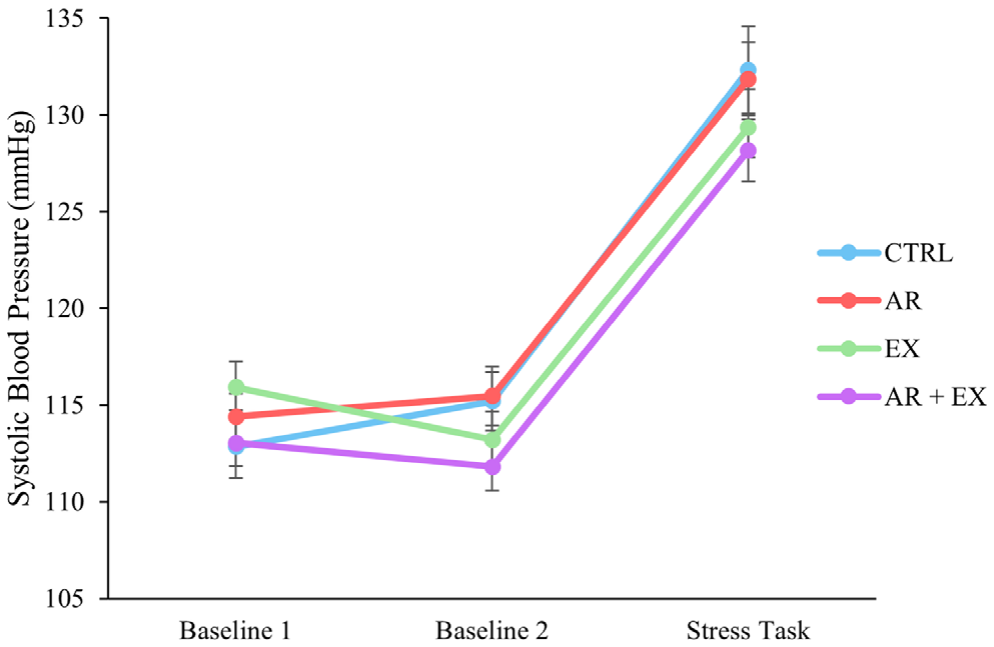
Mean systolic blood pressure by group and phase. Error bars represent standard error of the mean. The activity phase took place in between baseline 1 and baseline 2. AR, arousal reappraisal only group; AR + EX, combined arousal reappraisal and exercise group; CTRL, control group; EX, exercise only group; mmHg, millimeters of mercury.

A 4 group (CTRL, AR, EX, AR + EX) × 3 time (baseline 1, baseline 2, stress task) ANOVA was conducted to examine the impact of group on SBP responses to acute stress. There was a statistically significant group × time interaction, *F*(5.05, 387.39) = 2.77, *p* = 0.018, *η*_p_^2^ = 0.04, and a statistically significant main effect of time, *F*(1.68, 387.39) = 484.36, *p* < 0.001, *η*_p_^2^ = 0.68. The main effect of group was not statistically significant, *F*(3, 230) = 0.77, *p* = 0.510, *η*_p_^2^ = 0.01. From baseline 1 to baseline 2, SBP increased in the CTRL group (*p* = 0.032), but decreased in the EX group (*p* = 0.010). In all groups, SBP increased from baseline 2 to the stress task (*p*s < 0.001). There were no differences in SBP between the four groups during the stress task (*p*s ≥ 0.135). See Figure 4.

#### 3.4.2 Diastolic Blood Pressure

**FIGURE 5:**
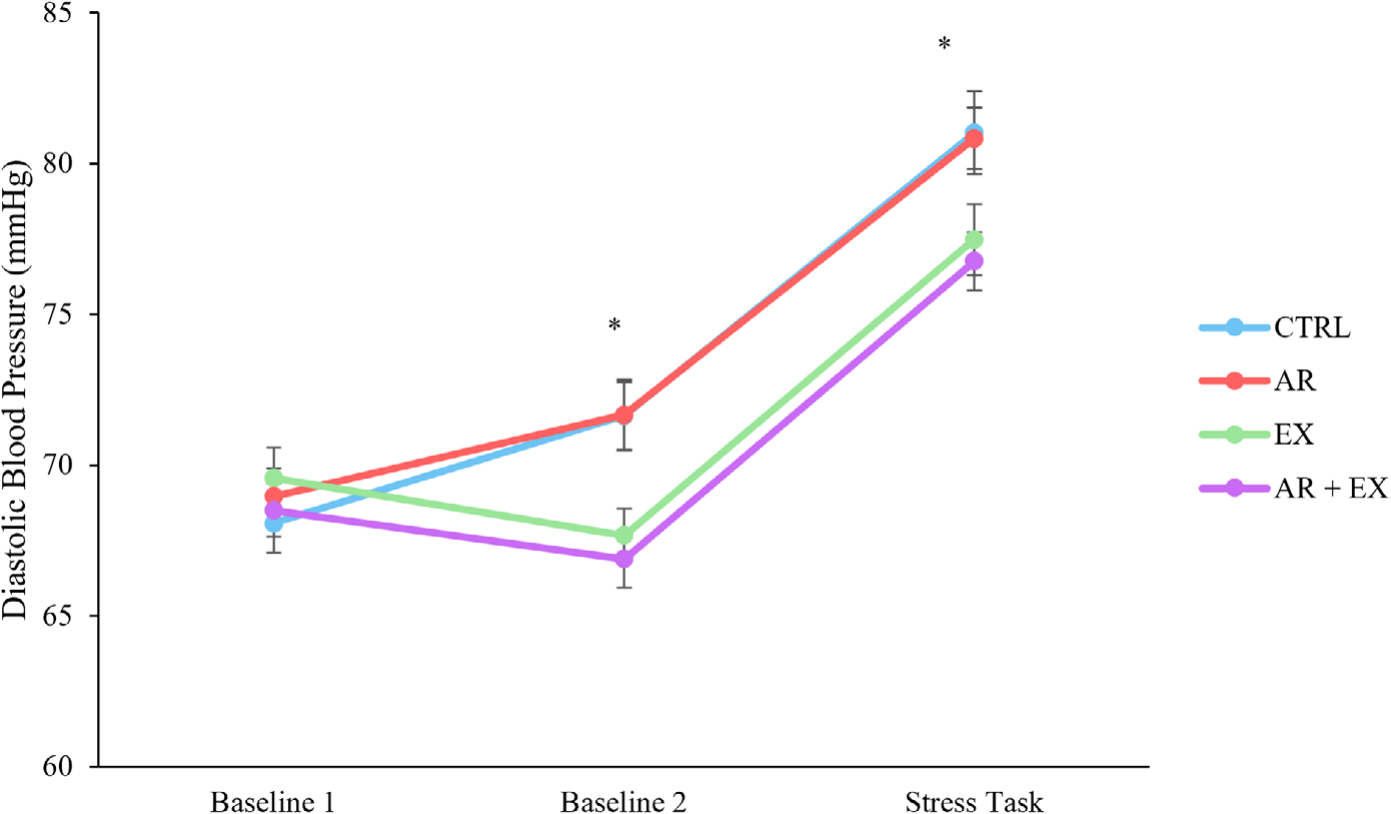
Mean diastolic blood pressure by group and phase. Error bars represent standard error of the mean. The activity phase took place in between baseline 1 and baseline 2. * indicates statistically significant differences between the exercise groups (EX, AR + EX) and non‐exercise groups (CTRL, AR). AR, arousal reappraisal only group; AR + EX, combined arousal reappraisal and exercise group; CTRL, control group; EX, exercise only group; mmHg, millimeters of mercury.

A 4 group (CTRL, AR, EX, AR + EX) × 3 time (baseline 1, baseline 2, stress task) ANOVA was conducted to examine the impact of group on DBP responses to acute stress. There was a statistically significant group × time interaction, *F*(5.79, 446.15) = 6.90, *p* < 0.001, *η*_p_^2^ = 0.08, and a statistically significant main effect of time, *F*(1.93, 446.15) = 369.69, *p* < 0.001, *η*_p_^2^ = 0.62. The main effect of group was not statistically significant, *F*(3, 231) = 2.62, *p* = 0.052, *η*_p_^2^ = 0.03. From baseline 1 to baseline 2, DBP increased in the non‐exercise groups (CTRL, AR; *p*s ≤ 0.001), decreased in the EX group (*p* = 0.042), and did not change in the AR + EX group (*p* = 0.120). In all groups, DBP increased from baseline 2 to the stress task (*p*s < 0.001). After accounting for multiple comparisons, there were no significant differences between the four groups during the stress task (Bonferroni corrected alpha = 0.004; *p*s ≥ 0.010). See Figure 5.

#### 3.4.3 Heart Rate

**FIGURE 6:**
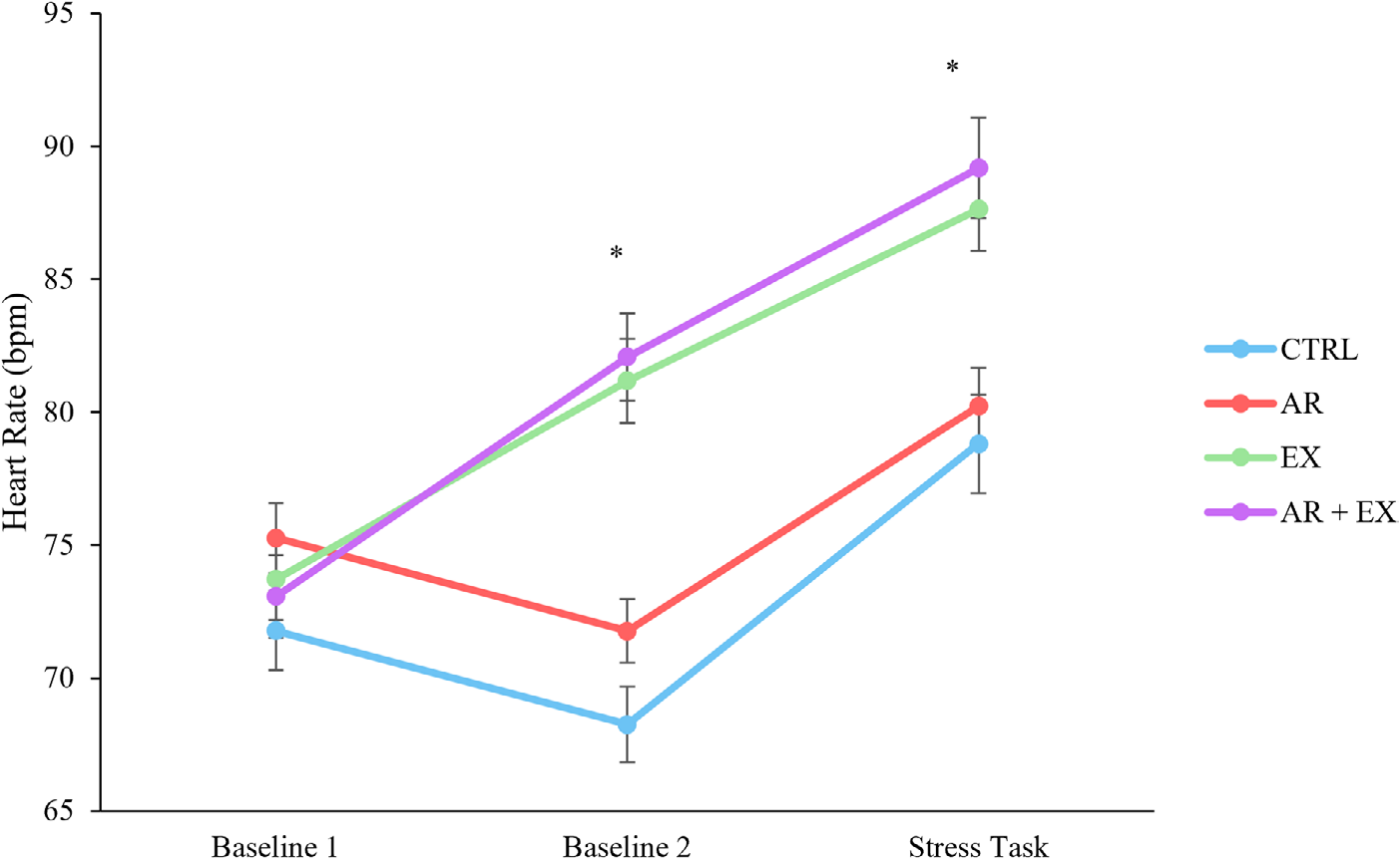
Mean heart rate by group and phase. Error bars represent standard error of the mean. The activity phase took place in between baseline 1 and baseline 2. * indicates statistically significant differences between the exercise groups (EX, AR + EX) and non‐exercise groups (CTRL, AR). AR, arousal reappraisal only group; AR + EX, combined arousal reappraisal and exercise group; bpm, beats per minute; CTRL, control group; EX, exercise only group.

A 4 group (CTRL, AR, EX, AR + EX) × 3 time (baseline 1, baseline 2, stress task) ANOVA was conducted to examine the impact of group assignment on HR responses to acute stress.^4^ There was a statistically significant group × time interaction, *F*(4.70, 350.79) = 29.23, *p* < 0.001, *η*_p_^2^ = 0.28. There was also a statistically significant main effect of time, *F*(1.57, 350.79) = 271.71, *p* < 0.001, *η*_p_^2^ = 0.55, and group, *F*(3, 224) = 7.06, *p* < 0.001, *η*_p_^2^ = 0.09. From baseline 1 to baseline 2, HR decreased for the non‐exercise groups (CTRL, AR; *p*s < 0.001), but increased for the exercise groups (EX, AR + EX; *p*s < 0.001). In all groups, HR increased from baseline 2 to the stress task (*p*s < 0.001). There was a statistically significant difference between the non‐exercise groups (CTRL, AR) and the exercise groups (EX, AR + EX) during the stress task (Bonferroni corrected alpha = 0.004; *p*s ≤ 0.002). See Figure 6.

### 3.5 Psychological Analyses

Mean values for each psychological variable are displayed in Table S2.

#### 3.5.1 Perceived Stress

**FIGURE 7:**
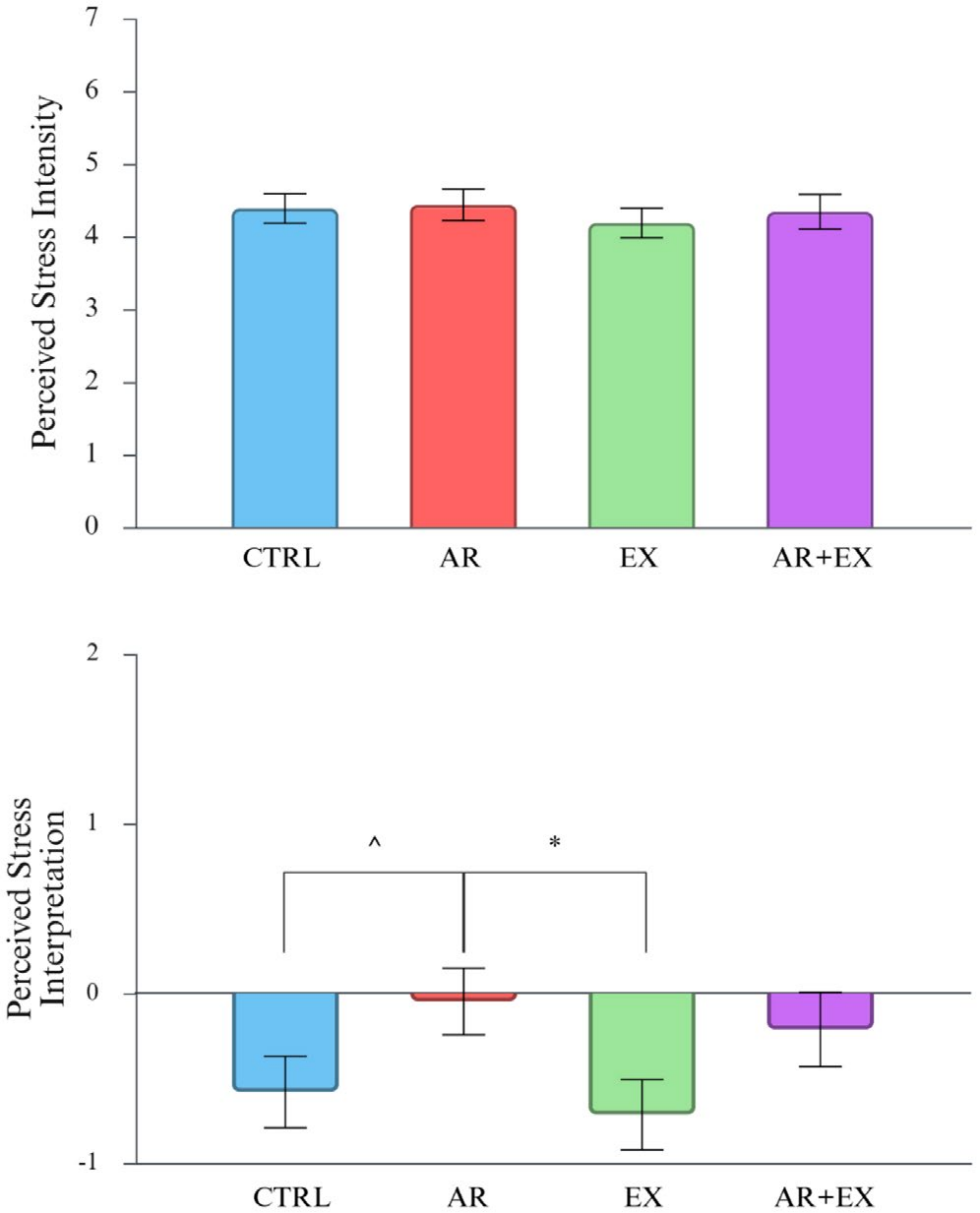
Mean perceived stress intensity and interpretation during the stress task by group. Error bars represent standard error of the mean. Differences between groups as indicated by post hoc contrasts are signified by: * *p* < 0.05, ^^^ *p* < 0.06. AR, arousal reappraisal only group; AR + EX, combined arousal reappraisal and exercise group; CTRL, control group; EX, exercise only group.

There were no statistically significant differences between groups in stressor‐evoked perceived stress intensity, *F*(3, 230) = 0.27, *p* = 0.848, *η*_p_^2^ = 0.003. However, there were differences between groups in the interpretation of stressor‐evoked perceived stress, *F*(3, 230) = 3.93, *p* = 0.009, *η*_p_^2^ = 0.05. Post hoc Bonferroni comparisons demonstrated that the AR group interpreted their stress as more facilitative than the EX group (*p* = 0.014). There was also a non‐significant trend indicating the AR group interpreted their stress as more facilitative than the CTRL group (*p* = 0.056). See Figure 7.

#### 3.5.2 Perceived Physiological Arousal

**FIGURE 8:**
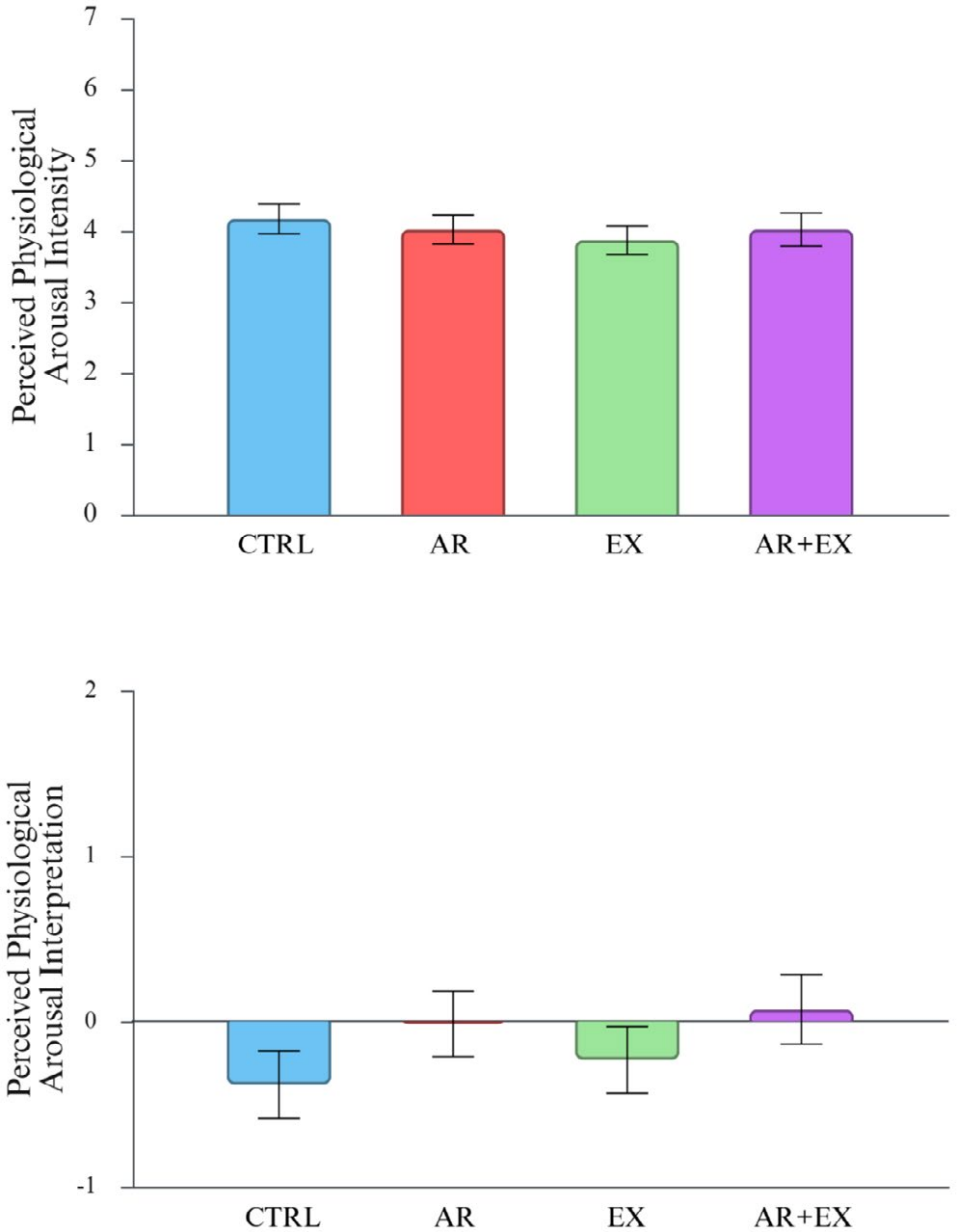
Mean perceived physiological arousal intensity and interpretation during the stress task by group. Error bars represent standard error of the mean. AR, arousal reappraisal only group; AR + EX, combined arousal reappraisal and exercise group; CTRL, control group; EX, exercise only group.

There were no statistically significant differences between groups in stressor‐evoked perceived physiological arousal intensity, *F*(3, 230) = 0.38, *p* = 0.769, *η*_p_^2^ = 0.01. Similarly, there were no statistically significant differences between groups in the stressor‐evoked perceived physiological arousal interpretation, *F*(3, 230) = 1.61, *p* = 0.188, *η*_p_^2^ = 0.02. See Figure 8.

### 3.6 Exploratory Moderation Analyses

To examine whether the effect of assigned group on the state psychological variables varied as a function of trait reappraisal, a moderation analysis using PROCESS Model 1 in SPSS was conducted (Hayes 2017). The multi‐categorical predictor was the assigned group (1 = CTRL, 2 = AR, 3 = EX and 4 = AR + EX), the moderator was trait reappraisal (continuous) and the dependent variables were the state psychological variables (continuous). For group assignment, the CTRL group was automatically coded as the reference category by PROCESS. Four moderated regression analyses were conducted (one per state psychological variable; see Tables S3 and S4). Trait reappraisal was not a statistically significant moderator in the relationship between group assignment and stressor‐evoked perceived stress intensity, interpretation of perceived stress or perceived physiological arousal intensity (all *p*s ≥ 0.258).

**FIGURE 9:**
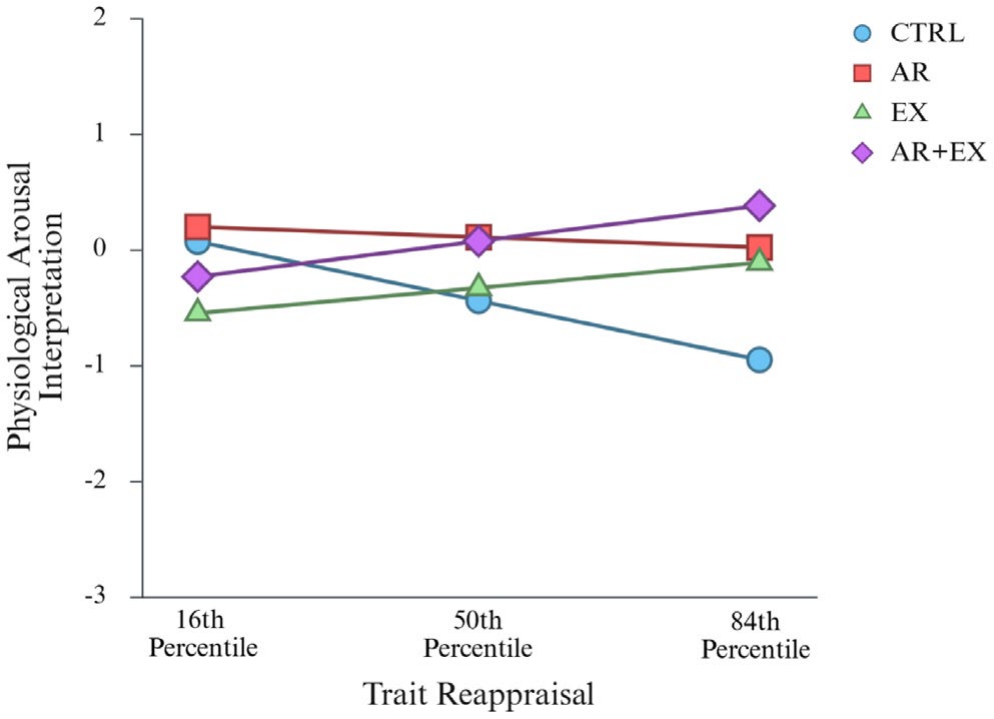
The moderating effect of trait reappraisal on the relationship between group assignment and stressor‐evoked physiological arousal interpretation. AR, arousal reappraisal only group; AR + EX, combined arousal reappraisal and exercise group; CTRL, control group; EX, exercise only group.

Trait reappraisal was a statistically significant moderator in the relationship between group assignment and interpretation of stressor‐evoked perceived physiological arousal. The overall model was significant, *F*(8, 226) = 2.95, *p* = 0.004, *R* ^2^ = 0.09. The interaction between group and trait reappraisal was also significant, *F*(3, 226) = 3.42, *p* = 0.018, Δ*R* ^2^ = 0.04. Significant interactions emerged between trait reappraisal and the EX group (*b* = 0.88, SE = 0.35, *t* = 2.42, *p* = 0.016) and trait reappraisal and the AR + EX group (*b* = 0.98, SE = 0.33, *t* = 3.00, *p* = 0.003), indicating that the relationship between group condition (EX and AR + EX vs. CTRL) and interpretation of perceived physiological arousal varied as a function of trait reappraisal. To probe the significant interactions, we examined the conditional effects of group assignment on the interpretation of perceived physiological arousal at different levels of the moderator. At low levels of trait reappraisal, there were no significant differences between the groups (*p*s ≥ 0.128). At high levels of trait reappraisal, the EX and AR + EX groups viewed their stressor‐evoked perceived physiological arousal as more facilitative than the CTRL group (*p*s ≤ 0.044). These results indicate trait reappraisal moderates the relationship between group and interpretation of perceived physiological arousal, such that individuals in the EX and AR + EX groups with higher trait reappraisal interpret their physiological arousal as more facilitative (see Figure 9).

## 4 Discussion

The present study examined the individual and combined effects of acute exercise and arousal reappraisal on psychological and cardiovascular responses to acute psychological stress. Contrary to the hypotheses, there were no differences between the four groups (CTRL, AR, EX, AR + EX) on stressor‐evoked cardiovascular responses, perceived stress intensity, perceived physiological arousal intensity, or interpretation of perceived physiological arousal. Interestingly, despite no group differences in stress intensity, the AR group had more positive interpretations of these stress levels. Lastly, moderation analyses examining the impact of trait reappraisal demonstrated that individuals in the EX and AR + EX groups with high levels of trait reappraisal interpreted their physiological arousal as more helpful than individuals in the CTRL group.

Previous literature demonstrated that arousal reappraisal instructions are associated with more positive views of stress (Beltzer et al. 2014; Gurera and Isaacowitz 2022; Huang et al. 2023; Sammy et al. 2017; Sharpe et al. 2024). The present study supports previous work; individuals in the AR group interpreted their stress more positively even though there were no differences between groups in self‐reported stress intensity. This aligns with the general ethos of arousal reappraisal which proposes that this process improves perceptions of stress, but does not eliminate feelings of stress (Jamieson, Crum, et al. 2018). Interestingly, and contrary to our hypothesis, the AR + EX group did not have significantly more positive interpretations of stress compared to the CTRL and EX groups. This result contrasts with the only previous study that combined arousal reappraisal and exercise and found that both the AR + EX and AR groups had more optimal stress responses (Jacquart et al. 2020). This discrepancy may reflect differences in participant characteristics (i.e., individuals with mild‐to‐moderate depressive symptoms in the previous study versus generally healthy young adults in the present study) and assessment of stress appraisals (i.e., the previous study measured this as a ratio of threat to challenge whereas the current study measured this as whether stress was facilitative or debilitative to performance). Additionally, the present study provided reappraisal for the AR + EX group during the rest period between exercise while the previous study provided reappraisal instructions during the acute exercise bout. Given these differences, it is difficult to determine which factor may be driving the difference in results. Future research should aim to systematically examine how these factors influence the impact of combining arousal reappraisal and exercise on stress interpretations.

Contrary to our hypotheses, there were no group differences in perceived physiological arousal intensity or interpretation. This result contrasts with previous work that has found arousal reappraisal is associated with more positive interpretations of physiological arousal (Ginty et al. 2022). However, limited work has explored the association between arousal reappraisal and physiological arousal, with more work exploring the relationship between arousal reappraisal and state anxiety (Moore et al. 2013; Sammy et al. 2017). As it has been proposed that arousal reappraisal alters stress responses by improving interpretations of physiological arousal, future research should continue to elucidate mechanisms through which arousal reappraisal may influence interpretations of physiological arousal and how this influence may be further altered by the inclusion of exercise.

While the current study found no group differences in the interpretation of perceived physiological arousal, moderation analyses revealed that among individuals with high trait reappraisal, those in the EX and AR + EX groups viewed their physiological arousal as more helpful than individuals in the CTRL group. This finding suggests that individuals who are more naturally inclined to reappraise may benefit the most from acute exercise. Previous work has found that acute exercise specifically enhances activation of regions of the brain associated with cognitive reappraisal (Zhang et al. 2021) and supports emotion regulation processes more broadly (Bernstein and McNally 2017; Edwards et al. 2017). It is possible that in those with elevated trait reappraisal abilities, acute exercise has an additive benefit to their already strong reappraisal abilities in coping with stress. However, more work is needed to understand the exact mechanisms underlying this association. Consistent with our findings, other work has demonstrated that the effectiveness of experimental conditions depends on individual's innate reappraisal abilities, with those high in trait reappraisal benefitting the most from arousal reappraisal (Mauersberger et al. 2018). In the AR only group, participants displayed similar positive interpretations of physiological arousal across all levels of trait reappraisal. Acute reappraisal instructions may have optimized interpretations regardless of trait reappraisal levels in the AR group.

To our knowledge, the current study was the first to simultaneously examine the impact of arousal reappraisal and exercise both separately and together on stressor‐evoked cardiovascular responses. Contrary to our hypotheses, there were no statistically significant differences between the groups for SBP nor DBP responses during stress, indicating neither exercise nor reappraisal attenuated BP during stress. For the exercise groups (EX, AR + EX), the null findings contrast with multiple systematic reviews and meta‐analyses demonstrating that acute exercise attenuates BP responses to stress (for reviews see: Chen et al. 2022; Hamer et al. 2006; Morava et al. 2024). These contrasting results may reflect differences in the exercise task, as few studies have used HIIT. Among those that have, one found attenuated BP responses to a cold pressor task following a bodyweight HIIT exercise session (Farah et al. 2021), whereas another found no differences in BP responses to a cold pressor task following HIIT cycling intervals (Meireles et al. 2020). However, for the reappraisal groups (AR, AR + EX), these null findings align with a recent meta‐analysis showing no effect of reappraisal on cardiovascular responses to acute stress (for review see: Liu et al. 2019). Aligned with our hypotheses, no statistically significant differences in stressor‐evoked heart rate responses were found. Although the HR responses to the stress task were relatively smaller than the BP responses, the magnitude of HR response to the stress task aligns with previous work using this stress task in a similar population (Tyra, Garner, and Ginty 2025). Given the variability in HIIT and arousal reappraisal protocols, future research should examine how certain characteristics (e.g., exercise intensity, duration of arousal reappraisal instructions) may impact cardiovascular responses to stress.

It should also be noted here that this study consisted of a singular laboratory visit. As participants only engaged in one instance of acute exercise, arousal reappraisal instructions or one combined session, it is possible that the benefits of arousal reappraisal and exercise can only be seen after repeated training sessions. Prior work has indicated that the influences of reappraisal training on emotions and behaviors can be seen after 1–3 weeks of training, although this work has been primarily conducted in clinical populations (Denny and Ochsner 2014; Kivity and Huppert 2016; Morris et al. 2015; Ng and Diener 2013). Exercise interventions may take longer to produce noticeable changes in stress responses, with most interventions lasting longer than three months (Atlantis et al. 2004; Churchill et al. 2022; Kettunen et al. 2015; Norris et al. 1992). As modifying beliefs about stress may require extended engagement, future research should employ longitudinal designs to investigate how arousal reappraisal and exercise may interact over time to influence psychophysiological responses to acute stress.

The results of the study should be interpreted in the context of the limitations. First, single‐item questions were used to assess the intensity and interpretations of perceived stress and physiological arousal. While single‐item measures can limit reliability, these items have been used in previous studies examining responses to acute psychological stress (e.g., Beevor et al. 2024; Cook et al. 2025; Ginty et al. 2022). Moreover, as arousal reappraisal focuses on reinterpreting, not minimizing or ignoring, stressor‐evoked physiological responses, these questions may provide valuable insight into specific ways arousal reappraisal alters psychological responses to stress. Second, the current study did not assess anticipatory stress responses. Previous work has found cardiovascular and psychological changes during anticipatory stress periods (e.g., Epstein and Clarke 1970; Feldman et al. 2004; Gramer and Sprintschnik 2008; Spacapan and Cohen 1983; Waugh et al. 2010). Nonetheless, treating speech preparation and delivery as one continuous stress task is common for research examining cardiovascular responses to acute psychological stress (e.g., Brindle et al. 2016; Tyra et al. 2020; Tyra, Garner, and Ginty 2025). Further, Jacquart et al. (2020) found no impact of arousal reappraisal and/or exercise on psychological responses in anticipation to stress. However, it may be important for future research to examine how arousal reappraisal and exercise may influence psychophysiological responses to both anticipatory stress and stress during the task. Third, although the sample included racial, ethnic and biological sex diversity, participants were relatively young (mean age = 18.93, SD = 1.10 years). Previous research has found older adults may benefit differently from arousal reappraisal and exercise (Gurera and Isaacowitz 2022; Pauly et al. 2019). Future research should examine how arousal reappraisal and acute exercise may influence stressor‐evoked psychophysiological responses differently by age. Additionally, it is possible that cardiorespiratory fitness may moderate the association between group and the outcome variables. However, the present study did not measure cardiorespiratory fitness across the entire sample. Future research should include a separate laboratory visit where measures of cardiorespiratory fitness are obtained (e.g., submaximal VO2 test; Ginty et al. 2026). Lastly, the cardiovascular analyses should be interpreted with caution as our findings indicate DBP was statistically significantly lower and HR was statistically significantly higher during the second baseline period (which occurred after the exercise and 30‐min recovery periods) compared to the first baseline period. Post‐exercise hypotension likely explains the significantly lower DBP values, with previous work finding DBP being reduced up to 45 min after exercise, which would coincide with the recovery period of the current study (Halliwill 2001). Similarly, prior work has found that heart rate activity remains elevated up to 60‐min post‐exercise (Alderman et al. 2007). Future research should consider utilizing a longer recovery period or exploring the impact of different recovery lengths on the relationship between acute exercise and psychophysiological responses to stress.

In conclusion, the present study examined how arousal reappraisal and exercise, both separately and together, influence psychophysiological responses to acute stress. While the present study found no impact of arousal reappraisal and exercise on cardiovascular responses to stress, it demonstrated that both arousal reappraisal and exercise may promote more adaptive interpretations of psychological responses to stress. However, there was no conclusive evidence that combining arousal reappraisal and acute exercise led to more adaptive stressor‐evoked responses than either training alone. Longitudinal research is needed to examine how repeated encounters with arousal reappraisal and exercise may promote more adaptive stressor‐evoked responses. Overall, these findings provide preliminary evidence that arousal reappraisal and exercise may be effective strategies for improving the interpretation of stress‐related psychological responses.

## Author Contributions

**Thomas A. Fergus:** writing – review and editing. **Sarah E. Williams:** conceptualization, validation, methodology, writing – review and editing. **Taryn E. Cook:** conceptualization, methodology, investigation, data curation, funding acquisition, project administration, formal analysis, visualization, writing – original draft. **Annie T. Ginty:** conceptualization, validation, resources, methodology, project administration, funding acquisition, formal analysis, supervision, writing – review and editing.

## Funding

A grant awarded to T.E.C. from the Department of Psychology and Neuroscience at Baylor University supported this project.

## Conflicts of Interest

The authors declare no conflicts of interest.

## Supporting information


**Table S1:** Means and standard deviations for cardiovascular measures for each phase for the total sample and by group.
**Table S2:** Means and standard deviations for psychological variables for the total sample and by group.
**Table S3:** Moderated regression models for group assignment predicting the intensity and interpretation of perceived stress moderated by trait reappraisal.
**Table S4:** Moderated regression models for group assignment predicting the intensity and interpretation of perceived physiological arousal moderated by trait reappraisal.

## Data Availability

The data that support the findings of this study are available from the corresponding author upon reasonable request.

## Endnotes

^1^
The 12 participants (7 female, 5 male, 58% white) were assigned to these groups: CTRL *n* = 2, EX *n* = 4, AR + EX *n* = 6.

^2^
Throughout the protocol, participants were given the same battery of questions repeatedly assessing the intensity and interpretation of their perceived physiological arousal and perceived stress. This battery of questions will be referred to as the state questionnaires moving forward.

^3^
Independent samples *t*‐tests on a subset of the data (*n* = 80) comparing the two exercise bikes revealed no statistically significant differences during exercise for average HR (*t*(78) = 0.11, *p* = 0.914) or average subjective rating of perceived exertion (*t*(78) = 0.37, *p* = 0.715).

^4^
Three participants' data were unusable for HR analyses only due to equipment malfunctions; thus, *n* = 235 for all HR analyses.

## References

[psyp70353-bib-0001] Abrantes, L. C. S. , N. de Souza de Morais , V. S. S. Gonçalves , et al. 2022. “Physical Activity and Quality of Life Among College Students Without Comorbidities for Cardiometabolic Diseases: Systematic Review and Meta‐Analysis.” Quality of Life Research 31: 1–30. 10.1007/s11136-021-03035-5. PMC8605778 34800221

[psyp70353-bib-0002] Agorastos, A. , and G. P. Chrousos . 2022. “The Neuroendocrinology of Stress: The Stress‐Related Continuum of Chronic Disease Development.” Molecular Psychiatry 27, no. 1: 502–513. 10.1038/s41380-021-01224-9. 34290370

[psyp70353-bib-0003] Aladro‐Gonzalvo, A. , G. A. Araya‐Vargas , A. Solera‐Herrera , J. Moncada‐Jiménez , and M. Machado‐Díaz . 2019. “Exercise Protects Cardiovascular Recovery From Stress in a Sample of Black Ethnicity Adolescents.” Gazzetta Medica Italiana, Archivio per le Scienze Mediche 178: 491–500. 10.23736/S0393-3660.18.03889-5.

[psyp70353-bib-0004] Alderman, B. L. , S. M. Arent , D. M. Landers , and T. J. Rogers . 2007. “Aerobic Exercise Intensity and Time of Stressor Administration Influence Cardiovascular Responses to Psychological Stress.” Psychophysiology 44, no. 5: 759–766. 10.1111/j.1469-8986.2007.00548.x. 17584185

[psyp70353-bib-0005] Alghamdi, A. S. , N. N. AlOyyna , and A. A. Alhusaini . 2025. “Physical Activities, Sedentary Behavior, Sleep Quality, and Quality of Life Among Female Medical Versus Nonmedical College Students: A Cross‐Sectional Study.” Medicine 104, no. 1: e41129. 10.1097/MD.0000000000041129. 40184114 PMC11709169

[psyp70353-bib-0006] American College of Sports Medicine . 2013. ACSM'S Guidelines for Exercise Testing and Prescription. Lippincott Williams & Wilkins. 10.1249/JSR.0b013e31829a68cf 23851406

[psyp70353-bib-0007] American Psychological Association . 2023. Stress in America 2023: A Nation Recovering From Collective Trauma. American Psychological Association.

[psyp70353-bib-0008] Ashton, L. M. , M. J. Hutchesson , M. E. Rollo , P. J. Morgan , and C. E. Collins . 2017. “Motivators and Barriers to Engaging in Healthy Eating and Physical Activity: A Cross‐Sectional Survey in Young Adult Men.” American Journal of Men's Health 11, no. 2: 330–343. 10.1177/1557988316680936. PMC5675273 27923963

[psyp70353-bib-0009] Atlantis, E. , C. Chow , A. Kirby , and M. Fiatarone Singh . 2004. “An Effective Exercise‐Based Intervention for Improving Mental Health and Quality of Life Measures: A Randomized Controlled Trial.” Preventive Medicine 39, no. 2: 424–434. 10.1016/j.ypmed.2004.02.007. 15226056

[psyp70353-bib-0010] Beevor, H. J. , A. T. Ginty , J. J. Veldhuijzen van Zanten , and S. E. Williams . 2024. “Mastery Imagery Ability Moderates the Relationship Between Heart Rate Reactivity to Acute Psychological Stress and Perceptions of Stress and Physiological Arousal.” Psychophysiology 61, no. 4: e14486. 10.1111/psyp.14486. 37973366

[psyp70353-bib-0011] Beltzer, M. L. , M. K. Nock , B. J. Peters , and J. P. Jamieson . 2014. “Rethinking Butterflies: The Affective, Physiological, and Performance Effects of Reappraising Arousal During Social Evaluation.” Emotion 14, no. 4: 761–768. 10.1037/a0036326. 24749642

[psyp70353-bib-0012] Benvenutti, M. J. , E. da Sliva Alves , S. Michael , D. Ding , E. Stamatakis , and K. M. Edwards . 2017. “A Single Session of Hatha Yoga Improves Stress Reactivity and Recovery After an Acute Psychological Stress Task—A Counterbalanced, Randomized‐Crossover Trial in Healthy Individuals.” Complementary Therapies in Medicine 35: 120–126. 10.1016/j.ctim.2017.10.009. 29154056

[psyp70353-bib-0013] Bernstein, E. E. , and R. J. McNally . 2017. “Acute Aerobic Exercise Helps Overcome Emotion Regulation Deficits.” Cognition and Emotion 31, no. 4: 834–843. 10.1080/02699931.2016.1168284. 27043051

[psyp70353-bib-0014] Bramley, P. , A. Easton , S. Morley , and R. Snaith . 1988. “The Differentiation of Anxiety and Depression by Rating Scales.” Acta Psychiatrica Scandinavica 77: 133–138. 10.1111/j.1600-0447.1988.tb05089.x. 3364199

[psyp70353-bib-0015] Brindle, R. C. , A. T. Ginty , A. Jones , et al. 2016. “Cardiovascular Reactivity Patterns and Pathways to Hypertension: A Multivariate Cluster Analysis.” Journal of Human Hypertension 30, no. 12: 755–760. 10.1038/jhh.2016.35. 27334523

[psyp70353-bib-0016] Chen, W. J. , A. F. Mat Ludin , and N. M. Farah . 2022. “Can Acute Exercise Lower Cardiovascular Stress Reactivity? Findings From a Scoping Review.” Journal of Cardiovascular Development and Disease 9, no. 4: 106. 10.3390/jcdd9040106. 35448082 PMC9029480

[psyp70353-bib-0017] Churchill, R. , K. Teo , L. Kervin , I. Riadi , and T. D. Cosco . 2022. “Exercise Interventions for Stress Reduction in Older Adult Populations: A Systematic Review of Randomized Controlled Trials.” Health Psychology and Behavioral Medicine 10, no. 1: 913–934. 36186892 10.1080/21642850.2022.2125874 PMC9518651

[psyp70353-bib-0018] Cohen, S. , P. J. Gianaros , and S. B. Manuck . 2016. “A Stage Model of Stress and Disease.” Perspectives on Psychological Science 11, no. 4: 456–463. 10.1177/1745691616646305. 27474134 PMC5647867

[psyp70353-bib-0019] Cohen, S. , D. Janicki‐Deverts , and G. E. Miller . 2007. “Psychological Stress and Disease.” Journal of the American Medical Association 298, no. 14: 1685–1687. 10.1001/jama.298.14.1685. 17925521

[psyp70353-bib-0020] Cook, T. E. , T. A. Fergus , D. A. Young , S. E. Williams , and A. T. Ginty . 2025. “Stressor‐Evoked Heart Rate, Perceived Physiological Arousal, and Anxiety Symptoms in Young Adults.” Journal of Affective Disorders 376: 454–462. 10.1016/j.jad.2025.02.011. 39922291 PMC13488176

[psyp70353-bib-0021] Craig, C. L. , A. L. Marshall , M. Sjöström , et al. 2003. “International Physical Activity Questionnaire: 12‐Country Reliability and Validity.” Medicine and Science in Sports and Exercise 35, no. 8: 1381–1395. 10.1249/01.MSS.0000078924.61453.FB. 12900694

[psyp70353-bib-0022] Crum, A. J. , P. Salovey , and S. Achor . 2013. “Rethinking Stress: The Role of Mindsets in Determining the Stress Response.” Journal of Personality and Social Psychology 104, no. 4: 716–733. 10.1037/a0031201. 23437923

[psyp70353-bib-0023] Dave, S. , M. Jaffe , and D. O'Shea . 2024. “Navigating College Campuses: The Impact of Stress on Mental Health and Substance Use in the Post COVID‐19 Era.” Current Problems in Pediatric and Adolescent Health Care 54, no. 5: 101585. 10.1016/j.cppeds.2024.101585. 38458900

[psyp70353-bib-0024] Denny, B. T. , and K. N. Ochsner . 2014. “Behavioral Effects of Longitudinal Training in Cognitive Reappraisal.” Emotion 14, no. 2: 425–433. 10.1037/a0035276. 24364856 PMC4096123

[psyp70353-bib-0025] Dickerson, S. S. , and M. E. Kemeny . 2004. “Acute Stressors and Cortisol Responses: A Theoretical Integration and Synthesis of Laboratory Research.” Psychological Bulletin 130, no. 3: 355–391. 10.1037/0033-2909.130.3.355. 15122924

[psyp70353-bib-0026] Ebben, W. , and L. Brudzynski . 2008. “Motivations and Barriers to Exercise Among College Students.” Journal of Exercise Physiology Online 11, no. 5: 1–11.

[psyp70353-bib-0027] Edwards, M. K. , R. E. Rhodes , and P. D. Loprinzi . 2017. “A Randomized Control Intervention Investigating the Effects of Acute Exercise on Emotional Regulation.” American Journal of Health Behavior 41, no. 5: 534–543. 10.5993/AJHB.41.5.2. 28760175

[psyp70353-bib-0028] Egloff, B. , S. C. Schmukle , L. R. Burns , and A. Schwerdtfeger . 2006. “Spontaneous Emotion Regulation During Evaluated Speaking Tasks: Associations With Negative Affect, Anxiety Expression, Memory, and Physiological Responding.” Emotion 6, no. 3: 356–366. 10.1037/1528-3542.6.3.356. 16938078

[psyp70353-bib-0029] Epel, E. S. , A. D. Crosswell , S. E. Mayer , et al. 2018. “More Than a Feeling: A Unified View of Stress Measurement for Population Science.” Frontiers in Neuroendocrinology 49: 146–169. 10.1016/j.yfrne.2018.03.001. 29551356 PMC6345505

[psyp70353-bib-0030] Epstein, S. , and S. Clarke . 1970. “Heart Rate and Skin Conductance During Experimentally Induced Anxiety: Effects of Anticipated Intensity of Noxious Stimulation and Experience.” Journal of Experimental Psychology 84, no. 1: 105–112. 5480915 10.1037/h0028929

[psyp70353-bib-0031] Farah, N. M. , A. D. Amran , and A. M. Che Muhamed . 2021. “Attenuation of Stress‐Induced Cardiovascular Reactivity Following High‐Intensity Interval Exercise in Untrained Males.” Journal of Sports Sciences 39, no. 24: 2755–2762. 10.1080/02640414.2021.1957294. 34323655

[psyp70353-bib-0032] Feldman, P. J. , S. Cohen , N. Hamrick , and S. J. Lepore . 2004. “Psychological Stress, Appraisal, Emotion and Cardiovascular Response in a Public Speaking Task.” Psychology & Health 19, no. 3: 353–368. 10.1080/0887044042000193497.

[psyp70353-bib-0033] Ge, Y. , S. Xin , D. Luan , et al. 2019. “Association of Physical Activity, Sedentary Time, and Sleep Duration on the Health‐Related Quality of Life of College Students in Northeast China.” Health and Quality of Life Outcomes 17: 1–8. 10.1186/s12955-019-1194-x. 31311564 PMC6636029

[psyp70353-bib-0034] Gerber, M. , S. Brand , C. Herrmann , F. Colledge , E. Holsboer‐Trachsler , and U. Pühse . 2014. “Increased Objectively Assessed Vigorous‐Intensity Exercise Is Associated With Reduced Stress, Increased Mental Health and Good Objective and Subjective Sleep in Young Adults.” Physiology & Behavior 135: 17–24. 10.1016/j.physbeh.2014.05.047. 24905432

[psyp70353-bib-0035] Gianaros, P. J. , and J. R. Jennings . 2018. “Host in the Machine: A Neurobiological Perspective on Psychological Stress and Cardiovascular Disease.” American Psychologist 73, no. 8: 1031–1044. 10.1037/amp0000232. 30394781 PMC6220680

[psyp70353-bib-0036] Gibala, M. J. 2007. “High‐Intensity Interval Training: A Time‐Efficient Strategy for Health Promotion?” Current Sports Medicine Reports 6, no. 4: 211–213. 10.1097/01.CSMR.0000306472.95337.e9. 17617995

[psyp70353-bib-0037] Ginty, A. T. , T. E. Kraynak , J. P. Fisher , and P. J. Gianaros . 2017. “Cardiovascular and Autonomic Reactivity to Psychological Stress: Neurophysiological Substrates and Links to Cardiovascular Disease.” Autonomic Neuroscience 207: 2–9. 10.1016/j.autneu.2017.03.003. 28391987 PMC5600671

[psyp70353-bib-0038] Ginty, A. T. , B. J. Oosterhoff , D. A. Young , and S. E. Williams . 2022. “Effects of Arousal Reappraisal on the Anxiety Responses to Stress: Breaking the Cycle of Negative Arousal Intensity and Arousal Interpretation.” British Journal of Psychology 113, no. 1: 131–152. 10.1111/bjop.12528. 34431517

[psyp70353-bib-0039] Ginty, A. T. , G. P. Trotman , A. G. Hogue , K. M. Knauft , J. J. C. S. Veldhuijzen van Zanten , and S. E. Williams . 2026. “The Effects of Acute Aerobic Exercise on Stressor‐Evoked Physiological and Psychological Responses.” International Journal of Psychophysiology 220: 113309. 10.1016/j.ijpsycho.2025.113309. 41397488 PMC13519942

[psyp70353-bib-0040] Gramer, M. , and E. Sprintschnik . 2008. “Social Anxiety and Cardiovascular Responses to an Evaluative Speaking Task: The Role of Stressor Anticipation.” Personality and Individual Differences 44, no. 2: 371–381. 10.1016/j.paid.2007.08.016.

[psyp70353-bib-0041] Griffin, S. M. , and S. Howard . 2021. “Instructed Reappraisal and Cardiovascular Habituation to Recurrent Stress.” Psychophysiology 58, no. 5: e13783. 10.1111/psyp.13783. 33538020

[psyp70353-bib-0042] Gross, J. J. , and O. P. John . 2003. “Individual Differences in Two Emotion Regulation Processes: Implications for Affect, Relationships, and Well‐Being.” Journal of Personality and Social Psychology 85, no. 2: 348–362. 10.1037/0022-3514.85.2.348. 12916575

[psyp70353-bib-0043] Gurera, J. W. , and D. M. Isaacowitz . 2022. “Arousal Reappraisal in Younger and Older Adults.” Psychology and Aging 37, no. 3: 350–356. 10.1037/pag0000674. 35084896

[psyp70353-bib-0044] Halliwill, J. R. 2001. “Mechanisms and Clinical Implications of Post‐Exercise Hypotension in Humans.” Exercise and Sport Sciences Reviews 29, no. 2: 65–70. 10.1097/00003677-200104000-00005. 11337825

[psyp70353-bib-0045] Hamer, M. , R. Endrighi , and L. Poole . 2012. “Physical Activity, Stress Reduction, and Mood: Insight Into Immunological Mechanisms.” In Psychoneuroimmunology: Methods and Protocols, 89–102. Springer. 10.1007/978-1-62703-071-7_5. 22933142

[psyp70353-bib-0046] Hamer, M. , A. Taylor , and A. Steptoe . 2006. “The Effect of Acute Aerobic Exercise on Stress Related Blood Pressure Responses: A Systematic Review and Meta‐Analysis.” Biological Psychology 71, no. 2: 183–190. 10.1016/j.biopsycho.2005.04.004. 15979232

[psyp70353-bib-0047] Hayes, A. F. 2017. Introduction to Mediation, Moderation, and Conditional Process Analysis: A Regression‐Based Approach. Guilford Publications.

[psyp70353-bib-0048] Herrmann, C. 1997. “International Experiences With the Hospital Anxiety and Depression Scale—A Review of Validation Data and Clinical Results.” Journal of Psychosomatic Research 42, no. 1: 17–41. 10.1016/s0022-3999(96)00216-4. 9055211

[psyp70353-bib-0049] Huang, Q. , H. Zhang , and R. Zhou . 2023. “Stress Reappraisal Improves the Autonomic Nervous System Response of Test Anxious Individuals: Evidence From Heart Rate Variability.” Neuroscience Letters 812: 137372. 10.1016/j.neulet.2023.137372. 37419306

[psyp70353-bib-0050] Jacquart, J. , S. Papini , Z. Freeman , J. B. Bartholomew , and J. A. J. Smits . 2020. “Using Exercise to Facilitate Arousal Reappraisal and Reduce Stress Reactivity: A Randomized Controlled Trial.” Mental Health and Physical Activity 18: 100324. 10.1016/j.mhpa.2020.100324.

[psyp70353-bib-0051] Jamieson, J. P. , A. E. Black , L. E. Pelaia , H. Gravelding , J. Gordils , and H. T. Reis . 2022. “Reappraising Stress Arousal Improves Affective, Neuroendocrine, and Academic Performance Outcomes in Community College Classrooms.” Journal of Experimental Psychology: General 151, no. 1: 197–212. 10.1037/xge0000893. 34292050

[psyp70353-bib-0052] Jamieson, J. P. , A. J. Crum , J. P. Goyer , M. E. Marotta , and M. Akinola . 2018. “Optimizing Stress Responses With Reappraisal and Mindset Interventions: An Integrated Model.” Anxiety, Stress, and Coping 31, no. 3: 245–261. 10.1080/10615806.2018.1442615. 29471669

[psyp70353-bib-0053] Jamieson, J. P. , E. J. Hangen , H. Y. Lee , and D. S. Yeager . 2018. “Capitalizing on Appraisal Processes to Improve Affective Responses to Social Stress.” Emotion Review 10, no. 1: 30–39. 10.1177/1754073917693085. 31178923 PMC6550483

[psyp70353-bib-0054] Jamieson, J. P. , W. B. Mendes , E. Blackstock , and T. Schmader . 2010. “Turning the Knots in Your Stomach Into Bows: Reappraising Arousal Improves Performance on the GRE.” Journal of Experimental Social Psychology 46, no. 1: 208–212. 10.1016/j.jesp.2009.08.015. 20161454 PMC2790291

[psyp70353-bib-0055] Jamieson, J. P. , W. B. Mendes , and M. K. Nock . 2013. “Improving Acute Stress Responses: The Power of Reappraisal.” Current Directions in Psychological Science 22, no. 1: 51–56. 10.1177/0963721412461500.

[psyp70353-bib-0056] Jamieson, J. P. , M. K. Nock , and W. B. Mendes . 2012. “Mind Over Matter: Reappraising Arousal Improves Cardiovascular and Cognitive Responses to Stress.” Journal of Experimental Psychology: General 141, no. 3: 417–422. 10.1037/a0025719. 21942377 PMC3410434

[psyp70353-bib-0057] Jamieson, J. P. , B. J. Peters , E. J. Greenwood , and A. J. Altose . 2016. “Reappraising Stress Arousal Improves Performance and Reduces Evaluation Anxiety in Classroom Exam Situations.” Social Psychological and Personality Science 7, no. 6: 579–587. 10.1177/1948550616644656.

[psyp70353-bib-0058] Kettunen, O. , T. Vuorimaa , and T. Vasankari . 2015. “A 12‐Month Exercise Intervention Decreased Stress Symptoms and Increased Mental Resources Among Working Adults–Results Perceived After a 12‐Month Follow‐Up.” International Journal of Occupational Medicine and Environmental Health 28, no. 1: 157–168. 10.13075/ijomeh.1896.00263. 26159956

[psyp70353-bib-0059] Kirschbaum, C. , K.‐M. Pirke , and D. H. Hellhammer . 1993. “The ‘Trier Social Stress Test’—A Tool for Investigating Psychobiological Stress Responses in a Laboratory Setting.” Neuropsychobiology 28, no. 1–2: 76–81. 10.1159/000119004. 8255414

[psyp70353-bib-0060] Kirschbaum, C. , S. Wüst , and D. Hellhammer . 1992. “Consistent Sex Differences in Cortisol Responses to Psychological Stress.” Biopsychosocial Science and Medicine 54, no. 6: 648–657. 10.1097/00006842-199211000-00004. 1454958

[psyp70353-bib-0061] Kivimäki, M. , A. Bartolomucci , and I. Kawachi . 2023. “The Multiple Roles of Life Stress in Metabolic Disorders.” Nature Reviews Endocrinology 19, no. 1: 10–27. 10.1038/s41574-022-00746-8. PMC10817208 36224493

[psyp70353-bib-0062] Kivity, Y. , and J. D. Huppert . 2016. “Does Cognitive Reappraisal Reduce Anxiety? A Daily Diary Study of a Micro‐Intervention With Individuals With High Social Anxiety.” Journal of Consulting and Clinical Psychology 84: 269–283. 10.1037/ccp0000075. 26795939

[psyp70353-bib-0063] LaManca, J. J. , A. Peckerman , S. A. Sisto , J. DeLuca , S. Cook , and B. H. Natelson . 2001. “Cardiovascular Responses of Women With Chronic Fatigue Syndrome to Stressful Cognitive Testing Before and After Strenuous Exercise.” Psychosomatic Medicine 63, no. 5: 756–764. 10.1097/00006842-200109000-00009. 11573024

[psyp70353-bib-0064] LeBouthillier, D. M. , and G. J. Asmundson . 2015. “A Single Bout of Aerobic Exercise Reduces Anxiety Sensitivity but Not Intolerance of Uncertainty or Distress Tolerance: A Randomized Controlled Trial.” Cognitive Behaviour Therapy 44, no. 4: 252–263. 10.1080/16506073.2015.1028094. 25874370

[psyp70353-bib-0065] Leow, S. , N. J. Beer , J. A. Dimmock , et al. 2021. “The Effect of Antecedent Exercise on the Acute Stress Response and Subsequent Food Consumption: A Preliminary Investigation.” Physiology & Behavior 229: 113256. 10.1016/j.physbeh.2020.113256. 33221392

[psyp70353-bib-0066] Liu, J. J. , N. Ein , J. Gervasio , and K. Vickers . 2019. “The Efficacy of Stress Reappraisal Interventions on Stress Responsivity: A Meta‐Analysis and Systematic Review of Existing Evidence.” PLoS One 14, no. 2: e0212854. 10.1371/journal.pone.0212854. 30811484 PMC6392321

[psyp70353-bib-0067] Mariano, I. M. , A. L. Amaral , P. A. B. Ribeiro , and G. M. Puga . 2022. “A Single Session of Exercise Reduces Blood Pressure Reactivity to Stress: A Systematic Review and Meta‐Analysis.” Scientific Reports 12, no. 1: 11837. 10.1038/s41598-022-15786-3. 35821393 PMC9276760

[psyp70353-bib-0068] Mauersberger, H. , A. Hoppe , G. Brockmann , and U. Hess . 2018. “Only Reappraisers Profit From Reappraisal Instructions: Effects of Instructed and Habitual Reappraisal on Stress Responses During Interpersonal Conflicts.” Psychophysiology 55, no. 9: e13086. 10.1111/psyp.13086. 29682755

[psyp70353-bib-0069] McFarlane, A. C. 2010. “The Long‐Term Costs of Traumatic Stress: Intertwined Physical and Psychological Consequences.” World Psychiatry 9, no. 1: 3–10. 10.1002/j.2051-5545.2010.tb00254.x. 20148146 PMC2816923

[psyp70353-bib-0070] Meireles, K. , T. Pecanha , A. R. Dias , et al. 2020. “Acute Effects of Moderate‐Intensity and High‐Intensity Exercise on Hemodynamic and Autonomic Reactivity to the Cold Pressor Test in Young Adults With Excess Body Weight.” Blood Pressure Monitoring 25, no. 2: 82–88. 10.1097/MBP.0000000000000422. 31833950

[psyp70353-bib-0071] Moore, L. J. , M. R. Wilson , S. J. Vine , A. H. Coussens , and P. Freeman . 2013. “Champ or Chump?: Challenge and Threat States During Pressurized Competition.” Journal of Sport and Exercise Psychology 35, no. 6: 551–562. 10.1123/jsep.35.6.551. 24334317

[psyp70353-bib-0072] Morava, A. , K. Dillon , W. Sui , E. Alushaj , and H. Prapavessis . 2024. “The Effects of Acute Exercise on Stress Reactivity Assessed via a Multidimensional Approach: A Systematic Review.” Journal of Behavioral Medicine 47, no. 4: 545–565. 10.1007/s10865-024-00470-w. 38468106

[psyp70353-bib-0073] Morris, R. R. , S. M. Schueller , and R. W. Picard . 2015. “Efficacy of a Web‐Based, Crowdsourced Peer‐to‐Peer Cognitive Reappraisal Platform for Depression: Randomized Controlled Trial.” Journal of Medical Internet Research 17, no. 3: e72. 10.2196/jmir.4167. 25835472 PMC4395771

[psyp70353-bib-0074] Murphy, J. J. , M. H. Murphy , C. MacDonncha , N. Murphy , A. M. Nevill , and C. B. Woods . 2017. “Validity and Reliability of Three Self‐Report Instruments for Assessing Attainment of Physical Activity Guidelines in University Students.” Measurement in Physical Education and Exercise Science 21, no. 3: 134–141. 10.1080/1091367X.2017.1297711.

[psyp70353-bib-0075] Ng, W. , and E. Diener . 2013. “Daily Use of Reappraisal Decreases Negative Emotions Toward Daily Unpleasant Events.” Journal of Social and Clinical Psychology 32, no. 5: 530–545. 10.1521/jscp.2013.32.5.530.

[psyp70353-bib-0076] Nguyen‐Michel, S. T. , J. B. Unger , J. Hamilton , and D. Spruijt‐Metz . 2006. “Associations Between Physical Activity and Perceived Stress/Hassles in College Students.” Stress and Health 22, no. 3: 179–188. 10.1002/smi.1094.

[psyp70353-bib-0077] Norris, R. , D. Carroll , and R. Cochrane . 1992. “The Effects of Physical Activity and Exercise Training on Psychological Stress and Well‐Being in an Adolescent Population.” Journal of Psychosomatic Research 36, no. 1: 55–65. 10.1016/0022-3999(92)90114-h. 1538350

[psyp70353-bib-0078] Obrist, P. A. 1981. “The Cardiac‐Somatic Relationship.” In Cardiovascular Psychophysiology: A Perspective, 47–81. Springer.

[psyp70353-bib-0079] Pauly, T. , V. I. Michalowski , U. M. Nater , et al. 2019. “Everyday Associations Between Older Adults' Physical Activity, Negative Affect, and Cortisol.” Health Psychology 38, no. 6: 494–501. 10.1037/hea0000743. 31008643

[psyp70353-bib-0080] Piao, X. , J. Xie , and S. Managi . 2024. “Continuous Worsening of Population Emotional Stress Globally: Universality and Variations.” BMC Public Health 24, no. 1: 3576. 10.1186/s12889-024-20961-4. 39716139 PMC11668040

[psyp70353-bib-0081] Rudland, J. R. , C. Golding , and T. J. Wilkinson . 2020. “The Stress Paradox: How Stress Can Be Good for Learning.” Medical Education 54, no. 1: 40–45. 10.1111/medu.13830. 31509282

[psyp70353-bib-0082] Sala, M. N. , P. Molina , B. Abler , H. Kessler , L. Vanbrabant , and R. Van De Schoot . 2012. “Measurement Invariance of the Emotion Regulation Questionnaire (ERQ). A Cross‐National Validity Study.” European Journal of Developmental Psychology 9, no. 6: 751–757.

[psyp70353-bib-0083] Sammy, N. , P. A. Anstiss , L. J. Moore , P. Freeman , M. R. Wilson , and S. J. Vine . 2017. “The Effects of Arousal Reappraisal on Stress Responses, Performance and Attention.” Anxiety, Stress, and Coping 30, no. 6: 619–629. 10.1080/10615806.2017.1330952. 28535726

[psyp70353-bib-0084] Schneiderman, N. , G. Ironson , and S. D. Siegel . 2005. “Stress and Health: Psychological, Behavioral, and Biological Determinants.” Annual Review of Clinical Psychology 1, no. 1: 607–628. 10.1146/annurev.clinpsy.1.102803.144141. PMC2568977 17716101

[psyp70353-bib-0085] Seery, M. D. 2013. “The Biopsychosocial Model of Challenge and Threat: Using the Heart to Measure the Mind.” Social and Personality Psychology Compass 7, no. 9: 637–653. 10.1111/spc3.12052.

[psyp70353-bib-0086] Sharpe, B. T. , O. Leis , L. Moore , et al. 2024. “Reappraisal and Mindset Interventions on Pressurised Esport Performance.” Applied Psychology 73, no. 4: 2178–2199. 10.1111/apps.12544.

[psyp70353-bib-0087] Smits, J. A. , A. C. Berry , D. Rosenfield , M. B. Powers , E. Behar , and M. W. Otto . 2008. “Reducing Anxiety Sensitivity With Exercise.” Depression and Anxiety 25, no. 8: 689–699. 10.1002/da.20411. 18729145

[psyp70353-bib-0088] Sothmann, M. S. 2006. “The Cross‐Stressor Adaptation Hypothesis and Exercise Training.” In Psychobiology of Physical Activity, 149–160. Human Kinetics.

[psyp70353-bib-0089] Sothmann, M. S. , J. Buckworth , R. P. Claytor , R. H. Cox , J. E. White‐Welkley , and R. K. Dishman . 1996. “Exercise Training and the Cross‐Stressor Adaptation Hypothesis.” Exercise and Sport Sciences Reviews 24, no. 1: 267–288. 8744253

[psyp70353-bib-0090] Souza‐Talarico, J. N. , N. Wan , S. Santos , et al. 2016. “Cross‐Country Discrepancies on Public Understanding of Stress Concepts: Evidence for Stress‐Management Psychoeducational Programs.” BMC Psychiatry 16, no. 1: 181. 10.1186/s12888-016-0886-6. 27260184 PMC4893292

[psyp70353-bib-0091] Spacapan, S. , and S. Cohen . 1983. “Effects and Aftereffects of Stressor Expectations.” Journal of Personality and Social Psychology 45, no. 6: 1243–1254. 10.1037//0022-3514.45.6.1243. 6663445

[psyp70353-bib-0092] Steptoe, A. , and M. Kivimäki . 2012. “Stress and Cardiovascular Disease.” Nature Reviews Cardiology 9, no. 6: 360–370. 10.1038/nrcardio.2012.45. 22473079

[psyp70353-bib-0093] Stubbs, B. , D. Vancampfort , S. Rosenbaum , et al. 2017. “An Examination of the Anxiolytic Effects of Exercise for People With Anxiety and Stress‐Related Disorders: A Meta‐Analysis.” Psychiatry Research 249: 102–108. 10.1016/j.psychres.2016.12.020. 28088704

[psyp70353-bib-0094] Szabo, A. , P. François , G. Boudreau , L. Côté , L. Gauvin , and P. Seraganian . 1993. “Psychophysiological Profiles in Response to Various Challenges During Recovery From Acute Aerobic Exercise.” International Journal of Psychophysiology 14, no. 3: 285–292. 10.1016/0167-8760(93)90042-n. 8340246

[psyp70353-bib-0095] Thomas, M. C. , and T. W. Kamarck . 2023. “Does Light Physical Activity Reduce Blood Pressure Responses to Laboratory Stressors?” Psychophysiology 60, no. 6: e14294. 10.1111/psyp.14294. 36991308

[psyp70353-bib-0096] Trotman, G. P. , J. J. Veldhuijzen van Zanten , J. Davies , C. Möller , A. T. Ginty , and S. E. Williams . 2019. “Associations Between Heart Rate, Perceived Heart Rate, and Anxiety During Acute Psychological Stress.” Anxiety, Stress, and Coping 32, no. 6: 711–727. 10.1080/10615806.2019.1648794. 31382769

[psyp70353-bib-0097] Turner, A. I. , N. Smyth , S. J. Hall , et al. 2020. “Psychological Stress Reactivity and Future Health and Disease Outcomes: A Systematic Review of Prospective Evidence.” Psychoneuroendocrinology 114: 104599. 10.1016/j.psyneuen.2020.104599. 32045797

[psyp70353-bib-0098] Tyra, A. T. , R. C. Brindle , B. M. Hughes , and A. T. Ginty . 2020. “Cynical Hostility Relates to a Lack of Habituation of the Cardiovascular Response to Repeated Acute Stress.” Psychophysiology 57, no. 12: e13681. 10.1111/psyp.13681. 32920855

[psyp70353-bib-0099] Tyra, A. T. , S.‐B. Garner , and A. T. Ginty . 2025. “Examining the Association Between Habitual Emotion Regulation Strategies and Cardiovascular Stress Reactivity Across Three Studies.” Biological Psychology 194: 108966. 10.1016/j.biopsycho.2024.108966. 39681253

[psyp70353-bib-0100] Tyra, A. T. , D. A. Young , and A. T. Ginty . 2023. “Emotion Regulation Tendencies and Cardiovascular Responses to Repeated Acute Psychological Stress.” International Journal of Psychophysiology 194: 112261. 10.1016/j.ijpsycho.2023.112261. 37914039

[psyp70353-bib-0101] Tyra, A. T. , D. A. Young , and A. T. Ginty . 2025. “The Impact of Experimentally Instructed Suppression on Cardiovascular Habituation During Repeated Stress.” Biological Psychology 196: 109007. 10.1016/j.biopsycho.2025.109007. 40054596

[psyp70353-bib-0102] Vaccarino, V. , and J. D. Bremner . 2024. “Stress and Cardiovascular Disease: An Update.” Nature Reviews Cardiology 21, no. 9: 603–616. 10.1038/s41569-024-01024-y. 38698183 PMC11872152

[psyp70353-bib-0103] Waugh, C. E. , S. Panage , W. B. Mendes , and I. H. Gotlib . 2010. “Cardiovascular and Affective Recovery From Anticipatory Threat.” Biological Psychology 84, no. 2: 169–175. 10.1016/j.biopsycho.2010.01.010. 20096747 PMC2875335

[psyp70353-bib-0104] Wheeler, E. A. , A. N. Santoro , and A. F. Bembenek . 2019. “Separating the ‘Limbs’ of Yoga: Limited Effects on Stress and Mood.” Journal of Religion and Health 58, no. 6: 2277–2287. 10.1007/s10943-017-0482-1. 28819762

[psyp70353-bib-0105] Whittaker, A. C. , A. Ginty , B. M. Hughes , A. Steptoe , and W. R. Lovallo . 2021. “Cardiovascular Stress Reactivity and Health: Recent Questions and Future Directions.” Psychosomatic Medicine 83, no. 7: 756–766. 10.1097/PSY.0000000000000973. 34297004

[psyp70353-bib-0106] Yang, A. X. , and C. K. Hsee . 2019. “Idleness Versus Busyness.” Current Opinion in Psychology 26: 15–18. 10.1016/j.copsyc.2018.04.015. 29705581

[psyp70353-bib-0107] Zhang, Y. , W. Shi , H. Wang , M. Liu , and D. Tang . 2021. “The Impact of Acute Exercise on Implicit Cognitive Reappraisal in Association With Left Dorsolateral Prefronta Activation: A fNIRS Study.” Behavioural Brain Research 406: 113233. 10.1016/j.bbr.2021.113233. 33737088

[psyp70353-bib-0108] Zigmond, A. S. , and R. P. Snaith . 1983. “The Hospital Anxiety and Depression Scale.” Acta Psychiatrica Scandinavica 67, no. 6: 361–370. 10.1111/j.1600-0447.1983.tb09716.x. 6880820

