# Supplementary material for: Examining the Impact of Acute Exercise and Arousal Reappraisal on Stressor‐Evoked Psychological and Cardiovascular Responses
Source: Psychophysiology. 2026 Jun 28;63(7):e70353. doi: 10.1111/psyp.70353 (PMC13310514; doi:10.1111/psyp.70353)
Supplement: Supplementary file 1 — Table S1: Means and standard deviations for cardiovascular measures for each phase for the total sample and by group. Table S2: Means and standard deviations for psychological variables for the total sample and by group. Table S3: Moderated regression models for group assignment predicting the intensity and interpretation of perceived stress moderated by trait reappraisal. Table S4: Moderated regression models for group assignment predicting the intensity and interpretation of perceived physiological arousal moderated by trait reappraisal. [file PSYP-63-e70353-s001.docx]

*Supplemental Material for*

**Examining the Impact of Acute Exercise and Arousal Reappraisal on Stressor-Evoked Psychological and Cardiovascular Responses**

Taryn E. Cook, M.A., Sarah E. Williams, Ph.D., Thomas A. Fergus, Ph.D., & Annie T. Ginty, Ph.D.

**Arousal Reappraisal Instructions**

Before the activity, AR and AR+EX participants read/listened to these instructions: “Physiological arousal refers to how activated your body’s biological systems are (e.g., heart rate, adrenaline). People often report increases in physiological arousal to stressful situations (e.g., taking an exam, giving a speech). People tend to feel these increases are harmful to their performance, but physiological arousal may actually be helpful.”

Halfway through the activity, AR+EX participants read/listened to these instructions: “Take a moment to notice your heart rate, a measure of your physiological arousal. Right now, your physiological arousal is increased in response to exercise.” Both AR and AR+EX participants read/listened to these instructions “Research has shown that physiological arousal does not hurt performance during stress and can even help performance” along with an accompanying illustration.

At the conclusion of the activity, AR+EX participants read/listened to these instructions: “Take a moment to notice your heart rate, a measure of your physiological arousal. Right now, your physiological arousal is increased in response to exercise.” Both AR and AR+EX participants read/listened to these instructions “One study found that people who had greater increases in heart rate did better on a standardized math exam” along with a graph illustrating the point.

Lastly, based on prior reappraisal research (Jamieson et al., 2010), before the stress task, all participants heard/read the following instructions: “The goal of this research is to examine how physiological arousal during a speech correlates with performance. Because it is normal for people to feel stressed or anxious during public speaking, the equipment will measure cardiovascular changes that indicate your current physiological arousal.” For participants in the reappraisal groups (AR, AR+EX), this statement was followed with additional reappraisal instructions (Jamieson et al., 2010): “Interestingly, people think that physiological arousal (like you just experienced during the exercise portion of this study) during public speaking will negatively impact their performance.​ However, recent research suggests that physiological arousal does not hurt performance during public speaking and can even help performance. ​People who feel aroused and anxious during a speech might actually do better! This means you shouldn’t feel concerned if you feel aroused or anxious while speaking today. ​If you find yourself feeling anxious, simply remind yourself that arousal can be helping you do well.” (The underlined portion in the instructions above was only given to the AR+EX group.)

After each set of instructions, participants (AR, AR+EX) were then asked to summarize what they just heard.

**Physical Activity Scoring**

For each type of PA (vigorous PA, moderate PA, and walking activity), participants reported how many days per week and minutes per day they engaged in each activity. The frequency and duration of these activities were used to calculate estimates of weekly metabolic equivalents (MET). This calculation was done using the MET values for each activity recommended by the IPAQ scientific group (vigorous PA MET value = 8.0, moderate PA MET value = 4.0, walking PA MET value = 3.3; Craig et al., 2003). For each participant, total MET minutes per week were calculated using the following equation: MET minutes = (days/week of vigorous PA * minutes/day of vigorous PA * 8.0) + (days/week of moderate PA * minutes/day of moderate PA * 4.0) + (days/week of walking * minutes/day of walking * 3.3). Then according to SF-IPAQ scoring recommendations, individuals were categorized into “high”, “moderate” or “low” levels of PA. High PA was defined as completing at least 3 vigorous activities per week and achieving at least 1500 MET-minutes/week OR completing at least 7 walking, moderate or vigorous activities per week and achieving at least 3000 MET-minutes/week. Moderate PA was defined as completing 3+ vigorous activities of at least 20 minutes per week OR completing 5+ moderate or walking activities of at least 30 minutes per week OR completing 5+ walking, moderate or vigorous activities per week and at least 500 MET-minutes/week.

**Table S1.** Means and Standard Deviations for Cardiovascular Measures for each Phase for the Total Sample and by Group.

| **Measure** | **Total** | **CTRL** | **AR** | **EX** | **AR+EX** |
| --- | --- | --- | --- | --- | --- |
| Baseline 1 SBP, mmHg, *M* (*SD*) | 114.05 (10.38) | 112.86 (12.47) | 114.40 (9.27) | 115.92 (10.38) | 113.04 (8.95) |
| Baseline 2 SBP, mmHg, *M* (*SD*) | 113.96 (11.02) | 115.21 (11.64) | 115.48 (11.68) | 113.22 (11.14) | 111.81 (9.20) |
| Stress Task SBP, mmHg, *M* (*SD*) | 130.43 (14.85) | 132.33 (17.30) | 131.86 (14.57) | 129.37 (14.96) | 128.17 (11.94) |
| Baseline 1 DBP, mmHg, *M* (*SD*) | 68.77 (7.35) | 68.07 (7.71) | 68.96 (7.28) | 69.57 (7.79) | 68.50 (6.62) |
| Baseline 2 DBP, mmHg, *M* (*SD*)* | 69.51 (8.23) | 71.64 (8.77) | 71.66 (9.07) | 67.67 (6.73) | 66.88 (6.99) |
| Stress Task DBP, mmHg, *M* (*SD*)* | 79.07 (8.95) | 81.02 (10.56) | 80.84 (7.90) | 77.49 (9.02) | 76.76 (7.28) |
| Baseline 1 HR, bpm, *M* (*SD*) | 73.52 (11.30) | 71.93 (11.38) | 75.47 (10.50) | 73.71 (11.57) | 72.82 (11.67) |
| Baseline 2 HR, bpm, *M* (*SD*)* | 75.65 (12.42) | 68.59 (10.50) | 71.77 (9.26) | 80.92 (12.08) | 81.88 (12.36) |
| Stress Task 1 HR, bpm, *M* (*SD*)* | 83.85 (13.52) | 79.11 (14.30) | 80.13 (1.27) | 87.49 (12.15) | 89.18 (13.71) |

*Note*: * indicates statistically significant differences between the exercise groups (EX, AR+EX) and non-exercise groups (CTRL, AR). CTRL: control group; AR: arousal reappraisal only group; EX: exercise only group; AR+EX: combined arousal reappraisal and exercise group; SBP = systolic blood pressure; DBP = diastolic blood pressure; HR = heart rate; mmHg = millimeters of mercury; bpm = beats per minute.

**Table S2.** Means and Standard Deviations for Psychological Variables for the Total Sample and by Group.

| **Measure** | **Total** | **CTRL** | **AR** | **EX** | **AR+EX** |
| --- | --- | --- | --- | --- | --- |
| Perceived Stress Intensity, *M* (*SD*) | 4.32 (1.64) | 4.38 (1.57) | 4.39 (1.66) | 4.18 (1.58) | 4.34 (1.78) |
| Perceived Stress Impact, *M* (*SD*)*^ | -0.38 (1.61) | -0.58 (1.63) | 0.00 (1.49) | -0.72 (1.61) | -0.21 (1.64) |
| Perceived Physiological Arousal Intensity, *M* (*SD*) | 4.01 (1.62) | 4.17 (1.62) | 3.98 (1.57) | 3.87 (1.57) | 4.02 (1.74) |
| Perceived Physiological Arousal Impact, *M* (*SD*) | -0.13 (1.55) | -0.38 (1.57) | 0.03 (1.50) | -0.23 (1.56) | 0.07 (1.57) |

*Note*: Statistically significant differences between groups as indicated by post-hoc contrasts are signified by: * AR significantly different from EX at *p* = .014; ^ AR different from CTRL at *p* = .056. CTRL: control group; AR: arousal reappraisal only group; EX: exercise only group; AR+EX: combined arousal reappraisal and exercise group

**Table S3.** Moderated Regression Models for Group Assignment Predicting the Intensity and Interpretation of Perceived Stress Moderated by Trait Reappraisal

|  | Perceived Stress Intensity | | | | | |  |  | |
| --- | --- | --- | --- | --- | --- | --- | --- | --- | --- |
|  | *B* | *SE* | *t* | *p* | *R*^2^ | CI Lower | | | CI Upper |
| Model |  |  |  |  | .082 |  | | |  |
| Intercept | 4.64 | 1.34 | 3.46 | .001 |  | 2.00 | | | 7.28 |
| AR | -1.56 | 1.83 | -0.85 | .394 |  | -5.17 | | | 2.04 |
| EX | -0.37 | 1.96 | -0.19 | .852 |  | -4.22 | | | 3.49 |
| AR+EX | -1.97 | 1.75 | -1.12 | .263 |  | -5.42 | | | 1.48 |
| Trait Reappraisal | -0.22 | 0.27 | -0.82 | .413 |  | -0.75 | | | 0.31 |
| AR x Trait Reappraisal | 0.31 | 0.36 | 0.87 | .389 |  | -0.40 | | | 1.03 |
| EX x Trait Reappraisal | 0.04 | 0.39 | .095 | .925 |  | -0.73 | | | 0.80 |
| AR+EX x Trait Reappraisal | 0.39 | 0.35 | 1.13 | .259 |  | -0.29 | | | 1.08 |
| Baseline Perceived Stress Intensity | 0.30 | 0.07 | 4.11 | <.001 |  | 0.15 | | | 0.44 |
|  | Perceived Stress Interpretation | | | | | |  |  | |
|  | *B* | *SE* | *t* | *p* | *R*^2^ | CI Lower | | | CI Upper |
| Model |  |  |  |  | .107 |  | | |  |
| Intercept | 0.86 | 1.28 | 0.37 | .505 |  | -1.67 | | | 3.39 |
| AR | -1.43 | 1.77 | -0.81 | .421 |  | -4.91 | | | 2.06 |
| EX | -2.48 | 1.90 | -1.31 | .193 |  | -6.22 | | | 1.26 |
| AR+EX | -1.69 | 1.70 | 0.99 | .323 |  | -5.04 | | | 1.66 |
| Trait Reappraisal | -0.29 | 0.26 | -1.13 | .258 |  | -0.80 | | | 0.22 |
| AR x Trait Reappraisal | 0.44 | 0.35 | 1.25 | .214 |  | -0.25 | | | 1.13 |
| EX x Trait Reappraisal | 0.47 | 0.38 | 1.26 | .210 |  | -0.27 | | | 1.22 |
| AR+EX x Trait Reappraisal | 0.42 | 0.34 | 1.25 | .211 |  | -0.24 | | | 1.09 |
| Baseline Perceived Stress Interpretation | 0.17 | 0.07 | 3.73 | <.001 |  | 0.13 | | | 0.42 |

*Note.* A more negative perceived stress interpretation indicates a more harmful interpretation. Group assignment was a multi-categorical predictor, where each group (AR, EX, AR+EX) were compared to the CTRL group. *B* = unstandardized regression coefficient. AR: arousal reappraisal only group; EX: exercise only group; AR+EX: combined arousal reappraisal and exercise group.

**Table S4**. Moderated Regression Models for Group Assignment Predicting the Intensity and Interpretation of Perceived Physiological Arousal Moderated by Trait Reappraisal

| . | Perceived Physiological Arousal Intensity | | | | | |  |  | |
| --- | --- | --- | --- | --- | --- | --- | --- | --- | --- |
|  | *B* | *SE* | *t* | *p* | *R*^2^ | CI Lower | | | CI Upper |
| Model |  |  |  |  | .046 |  | | |  |
| Intercept | 2.67 | 1.36 | 1.97 | .050 |  | .003 | | | 5.36 |
| AR | 0.30 | 1.84 | 0.16 | .870 |  | -3.32 | | | 3.92 |
| EX | 1.42 | 1.95 | 0.73 | .467 |  | -2.43 | | | 5.28 |
| AR+EX | -0.20 | 1.76 | -0.11 | .910 |  | -3.67 | | | 3.27 |
| Trait Reappraisal | 0.18 | 0.27 | 0.66 | .511 |  | -0.35 | | | 0.71 |
| AR x Trait Reappraisal | -0.10 | 0.37 | -0.27 | .789 |  | -0.82 | | | 0.62 |
| EX x Trait Reappraisal | -0.35 | 0.39 | -0.90 | .370 |  | -1.11 | | | 0.42 |
| AR+EX x Trait Reappraisal | 0.00 | 0.35 | 0.01 | .992 |  | -0.69 | | | 0.69 |
| Baseline Perceived Physiological Arousal Intensity | 0.21 | 0.07 | 2.91 | .004 |  | 0.07 | | | 0.35 |
|  | Perceived Physiological Arousal Interpretation | | | | | |  |  | |
|  | *B* | *SE* | *t* | *p* | *R*^2^ | CI Lower | | | CI Upper |
| Model |  |  |  |  | .095 |  | | |  |
| Intercept | 2.45 | 1.25 | 1.96 | .051 |  | -0.01 | | | 4.91 |
| AR | -1.98 | 1.73 | -1.15 | .252 |  | -5.38 | | | 1.42 |
| EX | -4.27 | 1.83 | -2.34 | .020 |  | -7.86 | | | -0.67 |
| AR+EX | -4.39 | 1.64 | -2.67 | .008 |  | -7.63 | | | -1.16 |
| Trait Reappraisal | -0.61 | 0.25 | -2.42 | .017 |  | -1.11 | | | -0.11 |
| AR x Trait Reappraisal | 0.51 | 0.34 | 1.47 | .142 |  | -0.17 | | | 1.18 |
| EX x Trait Reappraisal | 0.88 | 0.36 | 2.42 | .016 |  | 0.16 | | | 1.59 |
| AR+EX x Trait Reappraisal | 0.98 | 0.33 | 3.00 | .003 |  | 0.34 | | | 1.63 |
| Baseline Perceived Physiological Arousal Interpretation | 0.30 | 0.10 | 3.04 | .003 |  | 0.11 | | | 0.49 |

*Note.* A more negative perceived physiological arousal interpretation indicates a more harmful interpretation. Group assignment was a multi-categorical predictor, where each group (AR, EX, AR+EX) were compared to the CTRL group. *B* = unstandardized regression coefficient. AR: arousal reappraisal only group; EX: exercise only group; AR+EX: combined arousal reappraisal and exercise group.
